# Simulated data on bond of steel reinforcement in self-compacting concrete

**DOI:** 10.1016/j.dib.2020.105594

**Published:** 2020-04-21

**Authors:** Marian Sabău

**Affiliations:** Department of Civil and Environmental Engineering, Universidad de la Costa, 080020 Barranquilla, Colombia

**Keywords:** Self-compacting concrete, Bond model, Pull-out failure, Confining reinforcement, Relative rib area, Data analysis

## Abstract

In this paper, the data were simulated using the bond model, proposed in a previously published research article of the author, to predict the peak bond stress in self-compacting concrete (SCC). The parameters considered were the concrete compressive strength, the geometrical properties of the reinforcing bar, and the confining reinforcement. The data consisted of 500 simulations for various concrete grades (C12, C16, C20, C25, C30, C35, C40, C45, and C50) and reinforcing bar diameters (10, 12, 16, 20, and 25 mm). Exploratory data analysis (EDA) was conducted and descriptive statistics were used to analyse all the data and some subsets of interest. These data can be reused in a finite element analysis software to explicitly model the bond between SCC and reinforcing bars through point-to-point or surface-to-surface contact.

Specifications table

**Table:** 

Subject	Civil and Structural Engineering
Specific subject area	Bond of steel reinforcement in self-compacting concrete
Type of data	Tables and figures.
Data acquisition	The data were simulated using the bond model presented in a previously published research article of the author to predict the peak bond stress in SCC. Pull-out tests on bars with short anchorage lengths (l_d_ ≤ 5ϕ) and large relative rib areas (f_R_ ≥ 0.090) were simulated.
Data format	Raw and analysed.
Parameters for data collection	The parameters for the data were the concrete compressive strength, geometrical properties of the reinforcing bar, and confining reinforcement.
Description of data collection	The data consisted of 500 simulations for various concrete grades (C12, C16, C20, C25, C30, C35, C40, C45, and C50) and reinforcing bar diameters (10, 12, 16, 20, and 25 mm). The first 20 data correspond to the predicted values of the peak bond stress considering the parameters used in a previously published research article of the author.
Data source location	City: Barranquilla
	Country: Colombia
Data accessibility	Repository name: Mendeley Data
	Data identification number: 10.17632/4j8hcv62j3.3
	Direct URL to data: https://data.mendeley.com/datasets/4j8hcv62j3/3
Related research article	Sabău, M., Pop, I., & Oneţ, T. (2016). Experimental study on local bond stress-slip relationship in self-compacting concrete. Materials and Structures, 49(9), 3693–3711. https://doi.org/10.1617/s11527-015-0749-5

## Value of the Data

• These data can be used to determine the local bond stress–slip relationship in normal-strength SCC (fck ≤ 50 MPa).
• Civil engineers can use these data with other data on high-strength SCC (fck > 50 MPa) to develop the design expressions for the bond strength and anchorage lengths of reinforcing bars embedded in well-confined concrete in which pull-out failure may occur.
• These data can be used by other researchers to conduct similar experiments on high-strength SCC.
• These data may be relevant to the development of design expressions in standards regarding the anchorage lengths of reinforcing bars embedded in SCC.
• These data can be reused in a finite element analysis software to explicitly model the bond between SCC and reinforcing bars by point-to-point or surface-to-surface contact.

## 1 Data description

**Fig. 1:**
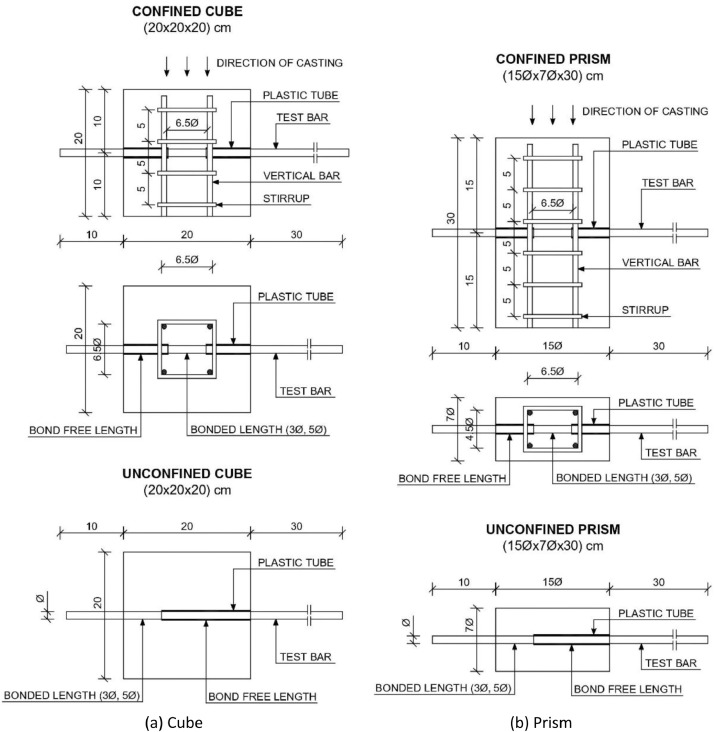
Characteristics of the specimens.

**Fig. 2:**
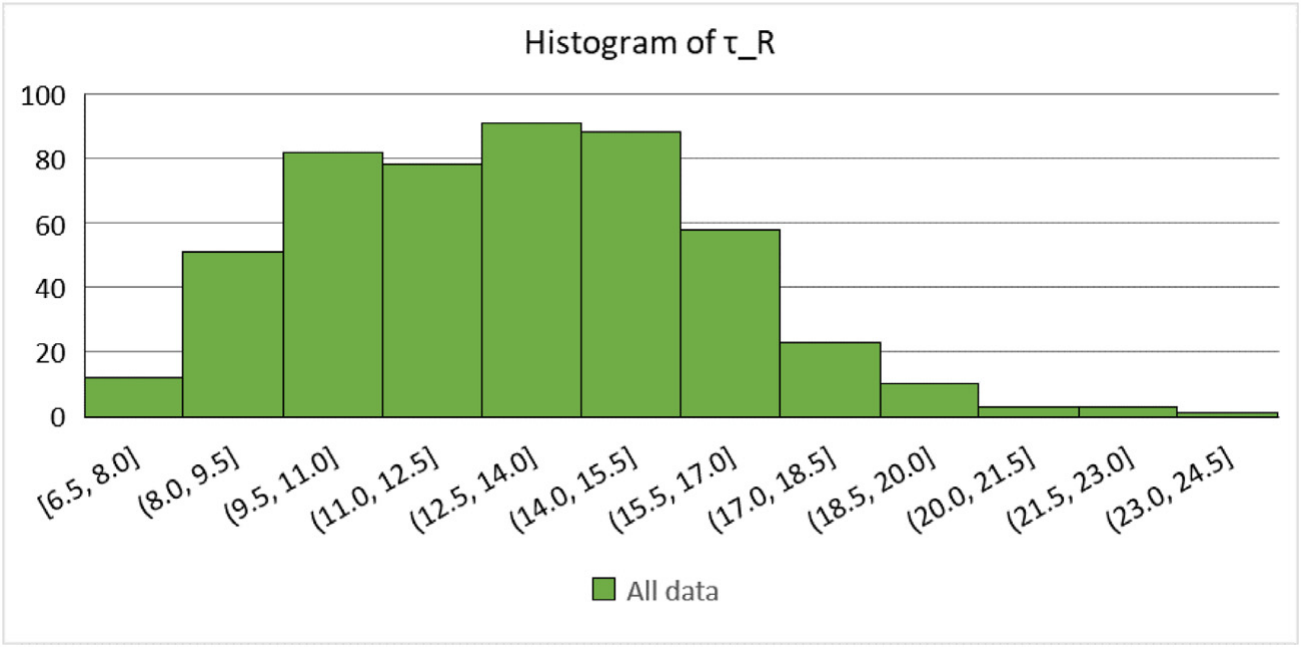
Distribution of the peak bond stress in the whole data.

**Fig. 3:**
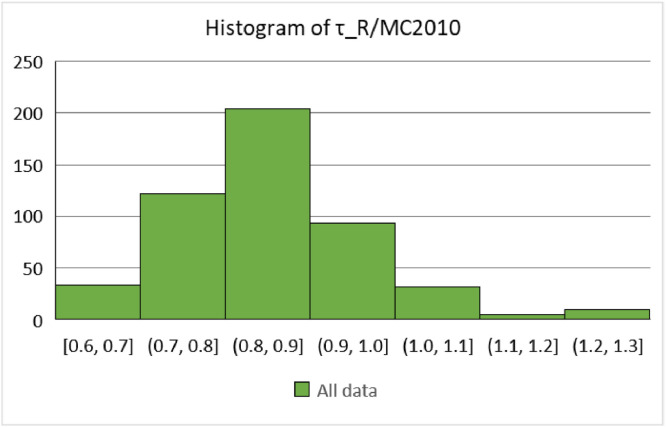
Distribution of the ratio, τ_R_/MC2010, in the whole data.

**Fig. 4:**
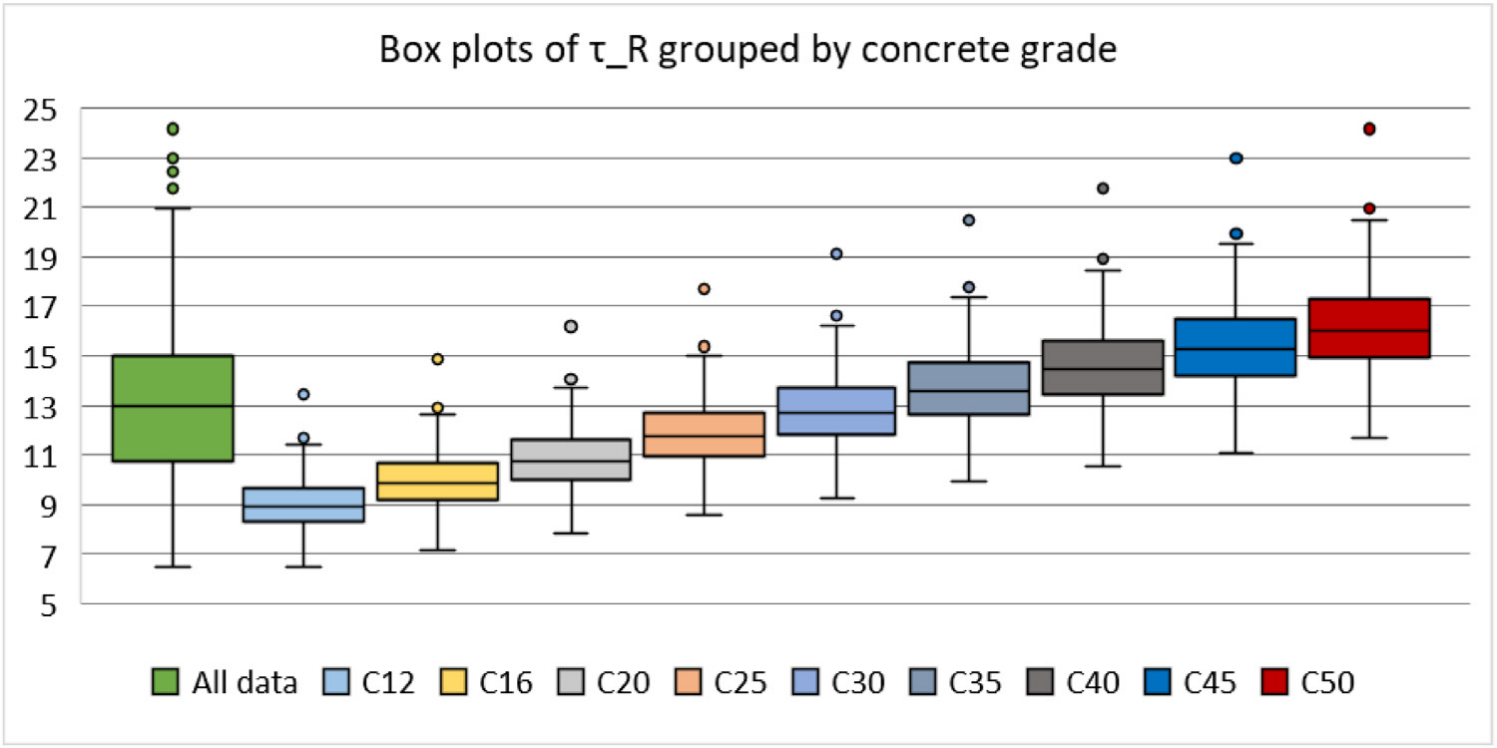
Distribution of the peak bond stress grouped by the concrete grade.

**Fig. 5:**
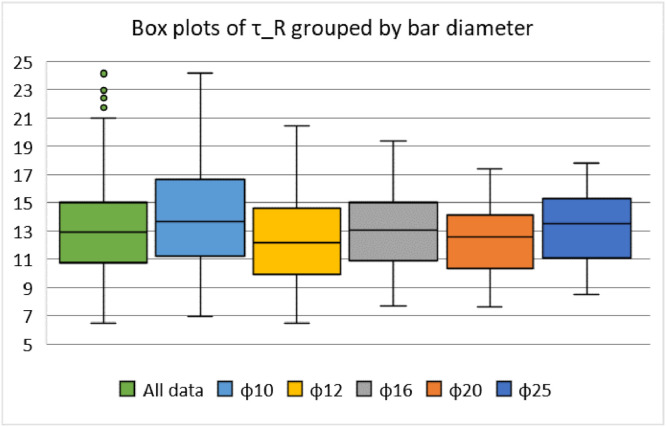
Distribution of the peak bond stress grouped by the bar diameter.

**Fig. 6:**
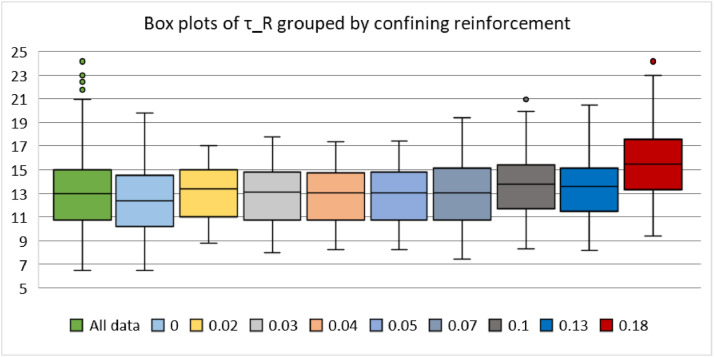
Distribution of the peak bond stress grouped by the confining reinforcement.

The data are available online in a public repository [1]. The data represent the simulated data obtained using the proposed analytical bond model for pull-out failure in a previous research article of the author to predict the peak bond stress in SCC [2]. The data consisted of 500 simulations for various concrete grades (C12, C16, C20, C25, C30, C35, C40, C45, and C50) and reinforcing steel bar diameters (10, 12, 16, 20, and 25 mm). Pull-out tests on bars with short anchorage lengths (l_d_ ≤ 5ϕ) and large relative rib areas (f_R_ ≥ 0.090) were simulated. The first 20 data correspond to the predicted values of the peak bond stress considering the parameters used in a previous research article of the author. The abbreviations used for the data are explained in Table 1. Fig. 1(a) and 1(b) display the geometric configurations of the specimens used for the simulation, cube, and prism specimens, respectively. Table 2 summarises the geometrical properties of the reinforcing bars anchored in these specimens. Figs. 2 and 3 display the histograms of the peak bond stress calculated using Eq. 1 (τ_R_), and the ratio of τ_R_ and the peak bond stress from fib Model Code 2010 [3], respectively. Table 3 summarises the statistical data for these histograms. Fig. 4, Fig. 5, Fig. 6 display the box plots of τ_R_ grouped by the concrete grade, bar diameter, and confining reinforcement, respectively. Table 4, Table 5, Table 6 summarise the statistical data for these box plots.

**Table 1:** Abbreviations used for the data.

Abbreviation	Meaning
f_cm	mean value of the concrete cylinder compressive strength (MPa)
ϕ	diameter of an anchored bar (mm)
c_min	minimum concrete cover (mm)
l_d	anchorage length of a bar (mm)
a_max	height of the transverse ribs of an anchored bar (mm)
c	distance between the transverse ribs of an anchored bar (mm)
f_R	relative rib area (-)
n_t	number of legs of the confining reinforcement crossing a potential splitting failure surface at a section (-)
A_st	cross-sectional area of one leg of a confining bar (mm^2^)
n_b	number of anchored bars (-)
s_t	longitudinal spacing of the confining reinforcement (-)
K_tr	density of the transverse reinforcement (-)
τ_R	peak bond stress calculated using Eq. 1 (MPa)
MC2010	peak bond stress from fib Model Code 2010 (MPa)
τ_R/MC2010	ratio of the peak bond stress calculated using Eq. 1 and the peak bond stress from fib Model Code 2010 (-)

**Table 2:** Geometrical properties of the anchored bars.

Anchored bar diameter, ϕ (mm)	Height of transverse ribs, a_max_ (mm)	Distance between the transverse ribs, c (mm)	Relative rib area, f_R_ (-)
10	0.60	6.4	0.094
12	0.70	7.8	0.090
16	0.76	7.5	0.101
20	0.95	9.5	0.100
25	1.80	16.6	0.108

**Table 3:** Summary of the statistical data of τ_R_ and τ_R_/MC2010 in the whole data.

	τ_R_	τ_R_/MC2010
Mean	12.99	0.83
SD	2.95	0.11
Min.	6.50	0.58
Max.	24.15	1.27
No. of data	500	500
Conf. level (95%)	0.26	0.01

**Table 4:** Summary of the statistical data for τ_R_ grouped by the concrete grade.

	C12	C16	C20	C25	C30	C35	C40	C45	C50
Mean	9.04	10.00	10.88	11.91	12.87	13.78	14.64	15.46	16.24
SD	1.24	1.37	1.49	1.63	1.77	1.89	2.01	2.12	2.23
Min.	6.50	7.18	7.82	8.56	9.25	9.90	10.51	11.10	11.67
Max.	13.45	14.86	16.18	17.71	19.14	20.48	21.76	22.98	24.15
No. of data	50	50	50	50	50	50	50	50	50
Conf. level (95%)	0.35	0.39	0.42	0.46	0.50	0.54	0.57	0.60	0.63

**Table 5:** Summary of the statistical data for τ_R_ grouped by the bar diameter.

	ϕ10	ϕ12	ϕ16	ϕ20	ϕ25
Mean	14.04	12.42	13.01	12.29	13.18
SD	3.72	3.03	2.66	2.37	2.46
Min.	6.93	6.50	7.69	7.60	8.54
Max.	24.15	20.48	19.35	17.43	17.17
No. of data	100	100	100	100	100
Conf. level (95%)	0.74	0.60	0.53	0.47	0.49

**Table 6:** Summary of the statistical data for τ_R_ grouped by the confining reinforcement.

	0	0.02	0.03	0.04	0.05	0.07	0.10	0.13	0.18
Mean	12.42	13.04	12.88	12.83	12.85	13.00	13.87	13.63	15.74
SD	2.78	2.42	2.52	2.50	2.52	2.87	3.33	3.23	3.91
Min.	6.50	8.75	7.94	8.22	8.21	7.44	8.30	8.18	9.35
Max.	19.80	17.00	17.77	17.33	17.43	19.35	20.96	20.48	24.15
No. of data	200	30	60	30	30	60	30	30	30
Conf. level (95%)	0.39	0.91	0.65	0.93	0.94	0.74	1.24	1.21	1.46

## 2 Experimental design, materials, and methods

A mathematical equation proposed in a previous research article of the author to predict the peak bond stress in SCC was used for the simulation. The equation describing the proposed bond model is based on multiple linear regression analysis of experimental data and is expressed in Eq. 1. All the parameters of this equation are explained in Table 1. Additional information about the bond model can be found in the related research article [2].

(1) $$\tau_{R}=\left(1.03\cdot \frac{\Phi }{l_{d}}+21\cdot \frac{a_{\text{max}}}{c}+0.10\cdot \frac{c_{\text{min}}}{\Phi }+2.55\cdot K_{\text{tr}}-1.14\right)\cdot {f_{\text{cm}}}^{0.55}$$

The parameters considered for the data simulation are presented below.

### 2.1 Concrete compressive strength

Normal-strength concrete is defined in fib Model Code 2010 [3] as concrete that has a characteristic compressive strength below 50 MPa (f_ck_ ≤ 50 MPa). In this simulation, all the concrete grades corresponding to this category were considered: C12, C16, C20, C25, C30, C35, C40, C45, and C50. The model used for the simulation (Eq. 1) considers the mean compressive strength (f_cm_); therefore, it was necessary to estimate this value from the characteristic compressive strength (f_ck_), because the concrete grades are defined in terms of this characteristic value. To estimate the mean strength from the characteristic strength, Eq. 5.1-1 from fib Model Code 2010 was used.

### 2.2 Reinforcing steel geometrical properties

The size and surface characteristics of the anchored bars are listed in Table 2. In total, five different sizes were considered. The relative rib area (f_R_) considered in the model for the simulation (Eq. 1) was estimated according to ACI 408R-03 [4] by adopting the ratio of the height of the transverse ribs (a_max_) and the distance between the transverse ribs (c). Only the bars with large relative rib areas were selected (f_R_ ≥ 0.090).

### 2.3 Specimen characteristics

The specimens used (cubes of 20 × 20 × 20 cm and prisms of 15ϕ × 7ϕ × 30 cm) simulated a confined beam–column connection. In total, 500 specimens were simulated: 200 cubes and 300 prisms. Fig. 1(a) and 1(b) display the characteristics of the specimens. Pull-out tests on deformed bars with short anchorage lengths (l_d_ ≤ 5ϕ) were considered. To avoid or control concrete splitting, specimens with a single bar and transverse reinforcement (K_tr_ > 0) were used. The bonded length was sufficiently long to reduce the scatter of the test data and sufficiently short to produce a uniform bond stress–slip.

### 2.4 Confining reinforcement

The confining reinforcement of the specimens represented the column vertical reinforcement (the four vertical bars of the reinforcing cages). The diameters of these bars were 6 and 8 mm. The density of the transverse reinforcement (K_tr_) was calculated for each configuration using Eq. 6.1-6 from fib Model Code 2010 [3]. The values obtained were 0, 0.02, 0.03, 0.04, 0.05, 0.07, 0.10, 0.13, and 0.18, respectively. In addition, unconfined specimens with the same characteristics as those of the confined ones but without confining reinforcement were used.

### 2.5 Data analysis

Exploratory data analysis (EDA) were conducted and descriptive statistics were used to analyse the whole data and some subsets of interest. Histograms and box plots were generated to obtain the distribution of the data, and the mean, standard deviation (SD), and minimum and maximum values were calculated to measure the central tendency and dispersion of the data. Fig. 2 displays the distribution of τ_R_ across the whole data, and Table 3 summarises the analysis. It was of interest to determine the difference between τ_R_ and the value for the peak bond stress from fib Model Code 2010 [3]. This analysis was conducted by calculating the ratio of the two parameters. In general, fib Model Code 2010 overestimates the peak bond stress, as can be noted from Fig. 3 and Table 3.

Three additional statistical analyses were conducted on some subsets of interest, as stated above. In the first analysis, the whole data were divided into nine subsets, with each subset representing the data for a particular concrete grade. The higher the compressive strength, the higher the τ_R_, as can be seen from Fig. 4 and Table 4. For the second analysis, the data were divided into five subsets, with each subset representing the data for a specific bar diameter. No remarkable trend in the data was observed in Fig. 5 and Table 5, which indicates that the bar diameter does not influence the peak bond stress significantly. In the final analysis, the data were divided again into nine subsets, with each subset representing the data for a particular confinement reinforcement density. A steady increase in τ_R_ beyond 10% transverse reinforcement density can be observed in Fig. 6 and Table 6.

## Conflict of Interest

The author declares that he has no known competing financial interests or personal relationships that could have appeared to influence the work reported in this paper.

## Appendix Supplementary materials

Image, application 1

Image, application 2
