# Supplementary material for: Simulated data on bond of steel reinforcement in self-compacting concrete
Source: Data Brief. 2020 Apr 21;30:105594. doi: 10.1016/j.dib.2020.105594 (PMC7191217; doi:10.1016/j.dib.2020.105594)
Supplement: Supplementary file 2 [file mmc2.pdf]

Materials and Structures
DOI 10.1617/s11527-015-0749-5

ORIGINAL ARTICLE

Experimental study on local bond stress-slip relationship in self-compacting concrete

Marian Sabău · Ioan Pop · Traian Oneţ

Received: 7 February 2015 / Accepted: 23 November 2015
© RILEM 2015

Abstract This paper summarizes the results of an experimental study on local bond stress-slip relationship of deformed reinforcing bars embedded in self-compacting concrete, considering the effect of concrete confinement by transverse reinforcement. In this study were cast 104 specimens by using self-compacting concrete and vibrated concrete. Pull-out tests were performed on deformed bars with short anchorage length ($l_d \leq 5\varnothing$). The parameters analyzed were the concrete type, the confining reinforcement, the bar diameter and the anchorage length of bar. The results of this study showed that the ultimate bond strength was higher for self-compacting concrete compared to vibrated concrete, and the slip corresponding to the ultimate bond strength was higher for vibrated concrete. Also, it has been observed higher ultimate bond strength for smaller bar diameter. In general a decreasing tendency of the ultimate bond strength with increased anchorage length of bar was found, yet with some exceptions. All the specimens confined with transverse reinforcement had a pull-out failure, while the unconfined specimens had a splitting failure, hence the presence of transverse reinforcement changed the type of bond failure from unfavorable (splitting), to a favorable one (pull-out). A new bond model for pull-out failure based on regression analysis of the experimental data from this study was proposed, considering the favorable effects that the confining reinforcement and relative rib area have on bond strength.

M. Sabău (✉)
Department of Civil Engineering, Universidad de la Costa, 080020 Barranquilla, Colombia
e-mail: marian.sabau@gmail.com

I. Pop
Magnel Laboratory for Concrete Research, Department of Structural Engineering, Ghent University, 9052 Ghent, Belgium

T. Oneţ
Department of Civil Structures, Technical University of Cluj-Napoca, 400027 Cluj-Napoca, Romania

Keywords Self-compacting concrete · Bond strength · Confining reinforcement · Pull-out test · Anchorage length · Relative rib area

1 Introduction

Self-compacting concrete (SCC) is a special type of concrete that is able to flow and consolidate under its own weight, completely filling the formwork even in the presence of a dense reinforcement, while maintaining uniformity, without the need for a mechanical compaction. In the late '80 s with the progress in the industry of additives for concrete, it was possible to increase the fluidity of the concrete making it self-compacting and sufficiently cohesive to prevent segregation. The concept of self-compacting concrete appeared and developed in Japan in 1988 [1], in order to achieve durable concrete structures by improving

Published online: 28 November 2015

the quality of casting and vibrating of conventional concrete. Self-compacting concrete offers economic and social benefits compared to vibrated concrete (VC), being able to provide superior quality for the surface of concrete elements after the removing of formwork. The concrete producers have shown that by using self-compacting concrete was achieved a reduction in the labor costs for the correction of surface imperfections by 25–75 % [2]. Removing the vibrating equipment improves the working environment in construction sites and precast concrete plants, reducing the risk of exposure of workers to noise and vibration. The constituent materials for self-compacting concrete are the same as those used in vibrated concrete: cement, additions, aggregates, admixtures, mixing water and possibly fibers. The quality of the materials for self-compacting concrete requires a higher level of control.

The bond between concrete and reinforcement is the physical phenomenon that allows longitudinal forces to be transferred from the reinforcement to the surrounding concrete in a reinforced concrete element. Because of this force transfer, the stress in a reinforcing bar changes along its length, as does the stress in the concrete. The most studies in the literature reported that the bond strength of SCC is higher than of VC. Gibbs and Zhu [3] found variations of the ultimate bond strength up to 32 % higher for SCC, but an increase of only 17 % for the normalized bond strength. Sonebi and Bartos [4] reported a variation of 16–40 % for the ultimate bond strength, and 7–26 % for the normalized bond strength. Sonebi et al. [5] evaluated a large number of pull-out tests to determine the bond between concrete and reinforcement, the results indicating a better bond strength for SCC compared to VC. Collepardi et al. [6] reported higher values for the normalized bond strength up to 70 %. Domone [7] made an analysis of more than 70 studies regarding the bond and hardened properties of SCC. He noticed that the bond strength between SCC and reinforcement is at least equal to VC, sometimes even higher. Almeida et al. [8] obtained for pull-out tests ultimate bond strengths that were higher for self-compacting concrete with 19 % for SCC1 compared to VC1, and 12 % for SCC2 compared to VC2. These results were for ∅10 bar diameter and 5∅ anchorage length of bar. In the case of beam tests they obtained ultimate bond strengths higher with 14 % for SCC1 compared to VC1, and equal ultimate bond strengths for SCC2 compared to VC2. These results were for ∅10 and ∅16 bar diameter, and 10∅ anchorage length of bar. Hossain and Lachemi [9] obtained for horizontal casting direction to the testing bar higher bond stress for FA SCC (self-compacting concrete with fly ash) as well as VMA SCC (self-compacting concrete with viscosity-modifying admixture) compared to VC. SC SCC (slag cement self-compacting concrete) developed lower bond stress compared to VC and other SCC mixtures. In the case of vertical casting direction to the testing bar they obtained higher normalized bond stresses for all SCC mixtures compared to VC. These results were for ∅25 bar diameter and 4∅ anchorage length of bar. Valcuende and Parra [10] found variations of 1–17 % for the ultimate bond strength, and 9–12 % for the normalized bond strength. Desnerk et al. [11] obtained higher bond strengths for SCC, as expected due to the higher compressive strength of SCC specimens compared to VC. Hassan et al. [12] obtained: normalized bond stresses slightly higher for SCC compared to VC at 3, 7, 14 and 28 days; higher ratio of the normalized bond stresses for SCC compared to VC in the top bars and late tested ages compared to the bottom bars and early tested ages; slightly higher bond stresses for both VC and SCC pullout specimens in the bottom bars compared to and middle bars at all ages. These results were for ∅20 bar diameter and 7.5∅ anchorage length of bar. Pop et al. [13] obtained higher ultimate bond strength up to 21 %, but for the normalized bond strength no difference between SCC and VC was noticed. On the other hand, there have been studies that reported higher ultimate bond strength for VC. For example, Schiessl and Zilch [14] and Almeida et al. [15], reported ultimate bond strength up to 15 % higher for VC. In the work presented by Rehm [16], he found that the bond strength tends to be a linear function of the relative rib area. Also, in a more recent study by Metelli and Plizzari [17], the authors found an increase in the bond strength with an increase of the relative rib area as follows: an increase of the relative rib area from 0.04 to 0.10 lead to an increase of the bond strength up to 40 %. The confining action of the concrete cover was studied by Darwin et al. [18] which found a linear increase of the anchorage capacity with the concrete cover and the clear distance between bars. According to fib MC2010 [19] concrete is considered well-confined when the concrete cover ≥5∅, and the clear distance between bars ≥10∅. It was found that

the size effects play a relevant role in the steel-to-concrete bond for smooth bars (Bazant et al. [20]), however for deformed bars embedded in well-confined concrete and for splitting-prone anchorages (Morita et al. [21]) these size effects are barely noticeable.

The objective of this study was to investigate the bond strength between concrete (SCC and VC) and reinforcement, especially the effect of concrete confinement with transverse reinforcement to improve the existing theoretical bond models, especially the one from fib MC2010 [19] for pull-out failure, proposing new equations for the calculation of the ultimate bond strength (τ_R), also making difference between SCC and VC.

2 Experimental program

In the experimental program were tested 104 specimens, 40 cubes and 64 prisms, by using SCC and VC as follows: series of three identical confined specimens and series of two identical unconfined specimens. The parameters analyzed were the concrete type (SCC and VC), the confining reinforcement (confined and unconfined specimens), the test bar diameter (∅10 and ∅12), and the anchorage length of bar (3∅ and 5∅).

2.1 Materials

The cement used was Portland cement CEM II/A-S 42.5R with ground granulated blast furnace slag (GGBFS), according to European standard EN 197-1 [22]. Natural aggregates were used, including sand 0–4 mm and two types of gravel, 4–8 and 8–16 mm, with relative densities of 2650 kg/m^3. In order to have an increased paste volume and a good cohesion, in the case of SCC, a limestone filler addition was used. For both concrete types, a high range water reducing admixture (HRWRA) based on modified polycarboxylic ether (PCE) polymers was used, in a dosage equal to 1.5 % of the amount of cement. The mixture proportions are summarized in Table 1.

The properties of the fresh concrete were determined immediately after the mixing procedure. The sieve segregation resistance test was used to determine the segregation resistance of SCC, according to EFNARC 2005 [23]. The surface of the fresh cast specimens was protected with a special resin-curing compound in order to prevent the loss of water. The specimens were demolded after 3 days and stored at a constant temperature of 20 ± 2 °C and a relative humidity of 95 ± 5 % until the time of testing. The compressive and flexural tensile strength were determined according to EN 12390-1 [24] at 28 days on cubes with sides of 150 mm for compression ($f_{c,cube}$), and prisms with a length of 550 mm and a height of 100 mm for flexural tensile strength ($f_{ct,fl}$). The flexural tensile strength was obtained by means of a four point bending test. The mean results of the fresh and hardened properties tests are presented in Table 2. It is noteworthy that the tensile strength of SCC was 30 % higher than that of VC, despite the fact that SCC and VC had the same compressive strength.

Table 1 Mixture proportions for SCC and VC

Materials	SCC	VC
Cement CEM II/A-S 42.5R (kg/m^3)	410	410
Limestone filler (kg/m^3)	190	–
Sand 0–4 mm (kg/m^3)	907	870
Gravel 4–8 mm (kg/m^3)	280	365
Gravel 8–16 mm (kg/m^3)	462	681
Superplasticizer (l/m^3)	6	3.2
Water (l/m^3)	175	175
Water/Cement	0.43	0.43
Water/Powder	0.29	–

In this research program, deformed bars were used for the test bars as well as for the bars of reinforcing cages. The test bars had the same rib pattern that consisted of parallel ribs with angles of 60° on one side and two alternating series of parallel ribs with angles of 50° and 70° on the other side. The diameter of the test bars used were ∅10 and ∅12, with the geometrical and mechanical properties presented in Table 3. The tested bars were horizontal at casting of the concrete specimens (Fig. 1a, b). For the reinforcing cages the diameter of the bars used were ∅6 and ∅8.

For each test bar were determined the following geometrical characteristics: the area of the projection of a single rib on the cross-section of the bar (F_R), the nominal diameter (∅), the distance between ribs (c) and the rib height (a_{max}), in order to obtain the relative rib area (f_R), according to EN ISO 15630-1 [25].

Table 2 Fresh and hardened properties of SCC and VC

Properties	SCC	Classification EFNARC 2005 [23]	VC
Slump flow (mm)	745	SF2	–
Slump (mm)	–	–	200
V funnel flow time (s)	6	VF1	–
Segregation resistance	9.7 %	SR2	–
Unit weight (kg/m^3)	2318	–	2367
$f_{c,cube}$ (MPa)	50.7	–	50.2
$f_{ct,fl}$ (MPa)	7.0	–	5.4

Table 3 Characteristics of test bars

Test bar diameter, ∅ (mm)	Steel grade	f_y (N/mm^2)	f_t (N/mm^2)	ε_{uk} (%)	Height of transverse ribs, a_{max} (mm)	Distance between the transverse ribs, c (mm)	Relative rib area, f_R [-]
10	S500	600	705	12	0.6	6.4	0.094
12	S500	560	656	14	0.7	7.8	0.090

2.2 Test set-up and testing procedure

Pull-out tests (Fig. 1c) were performed on deformed bars with only a fraction of their length in contact with confined concrete (upper part of Fig. 1a, b), that were similar to those of Eligehausen et al. [26]. In this way specimens with a single bar and heavy transverse reinforcement were tested to avoid concrete splitting or to keep it under control. The bonded length was long enough to reduce the scatter of test data and short enough to produce a uniform bond stress and slip.

The specimens used (cubes 20 × 20 × 20 cm, and prisms 15∅ × 7∅ × 30 cm), represented the confined beam-column connection. The confining reinforcement for the specimens (Table 4) represented the column reinforcement. The anchorage length of test bars was equal to three and five times the bar diameter (3∅ and 5∅). The casting direction was perpendicular to the longitudinal axis of the test bars. It has also been used unconfined specimens with the same characteristics as the confined ones but without confining reinforcement: cubes (20 × 20 × 20 cm) and prisms (15∅ × 7∅ × 30 cm) with ∅10 and ∅12 test bar diameter and anchorage length of test bars three and five times the bar diameter (3∅ and 5∅) as can be seen in the lower part of Fig. 1a, b. In total were cast 104 specimens in series of three identical confined specimens and series of two identical unconfined specimens for each parameter: the concrete type (SCC and VC), the specimen type (cube and prism), the test bar diameter (∅10 and ∅12), and the anchorage length of bar (3∅ and 5∅).

The minimum area of transverse reinforcement to be provided at anchorages to prevent a brittle mode of failure according to fib MC2010 [19] is calculated as follows (Eq. 1):

$$\sum A_{st,min} \geq 0.5 \cdot \sum A_s \quad (1)$$

where $\sum A_{st,min}$ is the minimum area of transverse reinforcement, $\sum A_s$ is the total cross-sectional area of all bars anchored at the section where confinement is provided by transverse reinforcement.

The tests were carried out at the age of 28 days. The applied force was measured during the test with a load cell (HBM U10 M 500KN). The slip of the unloaded end of the bar was recorded by using one linear variable differential transformer (LVDT) with a precision of 0.001 mm (HBM WA 10 mm). According to RILEM [27], the load rate was 50 N/sec for ∅10 test bars and 72 N/sec for ∅12 test bars, in order that the rate of increase of bond stress to be constant. The test was finished when pull-out failure occurred or splitting of the surrounding concrete was observed.

3 Test results

According to RILEM [27], the bond stress between reinforcement and concrete can be quantified by using Eq. 2 for uniform bond stress distribution over the

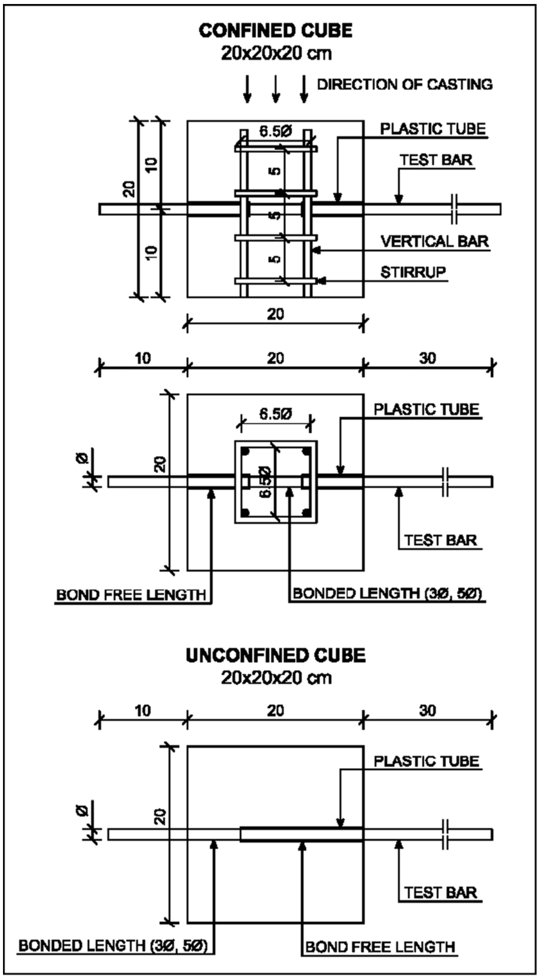

(a)

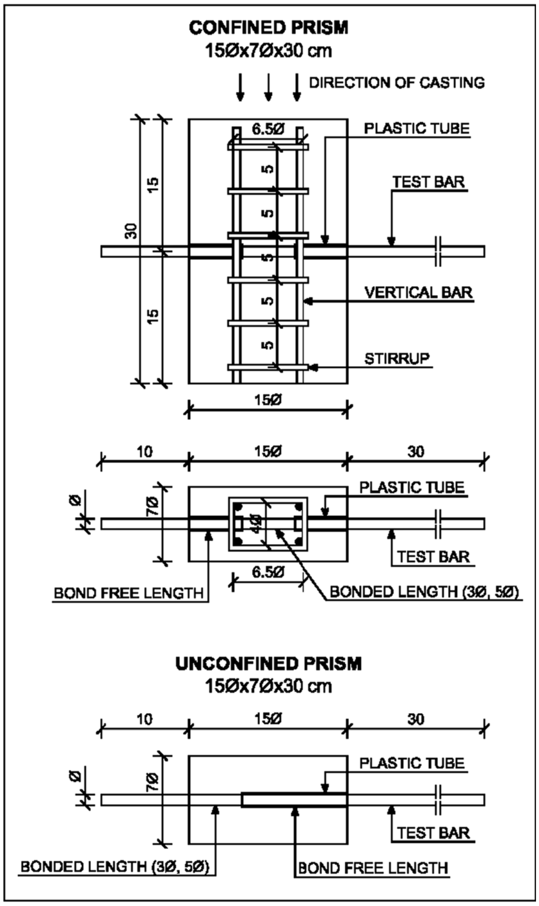

(b)

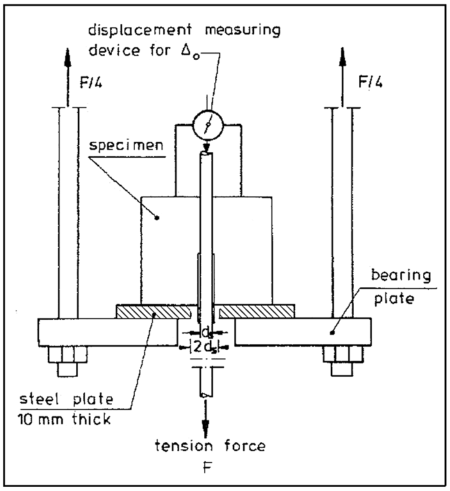

(c)

Fig. 1 Test setup characteristics. **a** Test specimen cube, **b** Test specimen prism, **c** Testing setup

embedment length, where F is the applied force, $\varnothing$ is the bar diameter and l_d is the anchorage length of bar.

$$\tau = \frac{F}{\pi \cdot \varnothing \cdot l_d} \quad (2)$$

The values of the local bond-stress slip relationship that were recorded are the ultimate bond strength (τ_R), the ultimate slip corresponding to the ultimate bond strength (s_u), the bond strength recorded at a slip of 0.01 mm ($\tau_{0.01}$), and the bond strength recorded at a slip of 0.1 mm ($\tau_{0.1}$). All these average values are presented in Table 5 for SCC and Table 6 for VC. The mode of failure and the standard deviation (SD) for three measurements in the case of confined specimens and two measurements in the case of unconfined specimens are also presented in the same tables. The values from these tables are the average of three measurements for identical confined specimens and the average of two measurements for identical unconfined specimens. Identical confined or unconfined specimens signify: identical concrete type (SCC or VC), identical specimen type (cube or prism), identical test bar diameter ($\varnothing$10 or $\varnothing$12) and identical anchorage length of bar (3$\varnothing$ or 5$\varnothing$).

All the comparisons that follow for each one of the parameters analyzed (Figs. 2, 3, 5 and 6) have been made between identical confined or unconfined specimens: identical concrete type (SCC or VC), identical specimen type (cube or prism), identical test bar diameter ($\varnothing$10 or $\varnothing$12), or identical anchorage length of bar (3$\varnothing$ or 5$\varnothing$), as was the case. One parameter was varied and all others were kept constant, in this way was avoided the mixing of the parameters.

3.1 Influence of the concrete type

In order to make a comparison between the two types of concrete, below are presented the bond stress-slip curves for confined cube specimens (Fig. 2a, b) and confined prism specimens (Fig. 2c, d), and the column type charts for ultimate bond strengths (Fig. 2e) and bond strengths recorded at a slip of 0.1 mm (Fig. 2f) for confined specimens. In this case the comparison has been made between identical SCC and VC confined specimens: identical specimen type (cube or prism), identical test bar diameter ($\varnothing$10 or $\varnothing$12), and identical anchorage length of bar (3$\varnothing$ or 5$\varnothing$).

As can be seen in Fig. 2e, the values recorded for the ultimate bond strength (τ_R), were higher for SCC up to 32 % ($\varnothing$10_3$\varnothing$) for confined cube specimens, and up to 25 % ($\varnothing$12_3$\varnothing$) for confined prism specimens. This is probably due to the increased amount of powder for SCC that reduces the risk of segregation which can lead to accumulation of water under the reinforcing bars. The values recorded for the ultimate slip (s_u), were higher for VC up to 34 % ($\varnothing$12_3$\varnothing$) for confined cube specimens. In the case of confined prism specimens the values obtained were contradictory, not always the VC specimens presented higher values (Tables 5 and 6). This behavior can be influenced by the 30 % higher tensile strength obtained for SCC (Table 2), which can influence the ultimate slip to a certain extent by increasing the capacity of the passive confinement, and by the increased amount of the paste used in the SCC mixture. The critical value of the bond strength that can be developed under service loads represents a fraction of the ultimate bond strength determined by

Table 4 Characteristics of the confining reinforcement

Specimen type	Test bar diameter, $\varnothing$ (mm)	Confining reinforcement	Minimum area of transverse reinforcement, $\sum A^{a}_{st,min}$ (cm^2)	Provided area of transverse reinforcement, $\sum A_{st}$ (cm^2)	Provided density of transverse reinforcement, K_{tr} (%)
Cube 20 × 20 × 20 cm	10	4 vertical bars $\varnothing$6	0.39	1.13	10
		4 vertical bars $\varnothing$8	0.39	2.01	18
	12	4 vertical bars $\varnothing$6	0.57	1.13	7
		4 vertical bars $\varnothing$8	0.57	2.01	13
Prism 15$\varnothing$ × 7$\varnothing$ × 30 cm	10	4 vertical bars $\varnothing$6	0.39	1.13	10
		4 vertical bars $\varnothing$8	0.39	2.01	18
	12	4 vertical bars $\varnothing$6	0.57	1.13	7
		4 vertical bars $\varnothing$8	0.57	2.01	13

[a] $\sum A_{st,min}$ is calculated from Eq. 1

Table 5 Experimental test results for SCC (average values)

Specimen type	Test bar diameter, ∅ (mm)	l_d	K_{tr} (%)	τ_R (MPa)	Values recorded							Mode of failure
					SD (MPa)	s_u (mm)	SD (mm)	$\tau_{0.01}$ (MPa)	SD (MPa)	$\tau_{0.1}$ (MPa)	SD (MPa)	
Cube 20 × 20 × 20 cm	10	3∅	18	21.75	3.20	0.74	0.10	6.83	2.50	15.02	1.52	Pull-out
			0	16.18	2.40	0.87	0.08	4.08	2.15	8.49	0.53	Pull-out
		5∅	10	19.43	2.27	0.65	0.05	7.05	1.27	12.65	0.41	Pull-out
			0	14.34	2.04	0.63	0.03	1.48	1.06	7.62	0.34	Pull-out
	12	3∅	13	20.62	2.86	0.83	0.40	6.70	0.44	14.33	0.66	Pull-out
			0	16.97	2.14	0.81	0.28	2.62	0.31	7.90	0.52	Pull-out
		5∅	7	18.60	1.86	1.49	0.11	5.26	0.35	9.86	1.05	Pull-out
			0	14.62	1.67	0.88	0.01	3.48	0.09	8.32	1.29	Pull-out
Prism 15∅ × 7∅ × 30 cm	10	3∅	18	15.29	2.57	1.14	0.34	4.49	0.27	10.35	1.23	Pull-out
			10	11.32	0.82	0.36	0.28	5.99	2.16	10.23	0.51	Pull-out
			0	13.00	0.74	0.31	0.21	6.71	0.24	11.76	0.47	Pull-out
		5∅	18	14.78	0.64	0.79	0.38	6.25	0.52	11.54	1.48	Pull-out
			10	15.39	1.20	0.88	0.24	6.48	0.44	11.89	1.22	Pull-out
			0	13.55	0.59	0.82	0.22	3.29	0.37	6.97	1.04	Pull-out
	12	3∅	13	16.28	0.59	0.60	0.24	8.70	0.28	13.64	0.35	Pull-out
			7	15.19	3.15	1.17	0.50	5.53	0.80	10.58	0.98	Pull-out
			0	10.12	0.52	0.67	0.20	3.63	0.23	4.86	0.32	Splitting
		5∅	13	13.14	0.96	1.67	0.44	6.31	0.89	9.32	0.51	Pull-out
			7	12.24	2.99	0.62	0.56	6.69	0.53	10.25	0.81	Pull-out
			0	8.85	0.86	0.08	0.39	6.17	0.48	8.82	0.45	Splitting

pull-out test. This critical value is expressed according to the slip of the free end of the reinforcing bar in concrete. The slip affects the crack width and the stiffness of reinforced concrete elements. In the literature [28] is considered the value 0.1 mm for the slip of the free end of the reinforcing bar as the slip corresponding to loads at service levels. In this study, the values obtained for the bond strength recorded at a slip of 0.1 mm ($\tau_{0.1}$), were higher for SCC up to 54 % (∅12_3∅) for confined cube specimens, and up to 117 % (∅12_3∅) for confined prism specimens (Fig. 2f). This is probably due to the lower porosity of the SCC compared with VC. For low level of loads ($\tau_{0.01}$), were recorded higher values for SCC up to 167 % (∅10_3∅) for confined cube specimens, and up to 266 % (∅12_3∅) for confined prism specimens (Tables 5 and 6). This trend of increase in stress for a slip of 0.01 mm in the case of SCC can be influenced by the compaction of the concrete under the reinforcing bars; this compaction in the case of SCC tends to be better. In the previous investigation at Technical University of Cluj-Napoca on bond behavior between steel reinforcement and unconfined SCC by Pop et al. [13], the ultimate bond strength (τ_R) was higher for SCC (up to 21 %) for compressive strengths around 50 MPa. The ultimate slip (s_u) corresponding to the ultimate bond strength was significantly higher for SCC (up to 141 %). For a slip of 0.01 mm SCC presented higher bond strength ($\tau_{0.01}$) compared to VC (up to 28 %). This improved bond behavior for SCC is confirmed by this new experimental study on confined SCC with similar compressive strength (f_c = 50.7 MPa).

Taking into account all these observations and the bond stress-slip curves for confined cube specimens (Fig. 2a, b), as well as for confined prism specimens (Fig. 2c, d), it can be concluded that the overall bond behavior was better for SCC compared to VC, for all bar diameters (∅10, ∅12) and all anchorage lengths of bar (3∅, 5∅).

3.2 Influence of the confining reinforcement

In order to analyze the influence of confining reinforcement, below are presented the bond stress-

Table 6 Experimental test results for VC (average values)

Specimen type	Test bar diameter, ∅ (mm)	l_d	K_{tr} (%)	Values recorded								Mode of failure
				τ_R (MPa)	SD (MPa)	s_u (mm)	SD (mm)	$\tau_{0.01}$ (MPa)	SD (MPa)	$\tau_{0.1}$ (MPa)	SD (MPa)	
Cube 20 × 20 × 20 cm	10	3∅	18	16.50	2.95	1.00	0.30	2.56	0.90	10.07	2.32	Pull-out
			0	12.03	0.86	0.68	0.27	0.89	0.84	7.31	2.16	Pull-out
		5∅	10	16.53	0.79	0.84	0.21	2.93	0.96	8.76	2.57	Pull-out
			0	12.54	0.73	0.77	0.18	0.89	0.93	5.15	2.35	Pull-out
	12	3∅	13	16.81	1.04	1.11	0.06	3.54	0.65	9.28	1.96	Pull-out
			0	8.93	0.91	0.56	0.05	3.10	0.57	5.15	1.73	Pull-out
		5∅	7	17.41	0.86	1.45	0.07	3.42	1.49	9.10	1.78	Pull-out
			0	10.63	0.74	0.78	0.04	1.73	1.35	6.22	1.64	Pull-out
Prism 15∅ × 7∅ × 30 cm	10	3∅	18	13.03	2.38	0.64	0.23	3.11	1.77	8.28	2.05	Pull-out
			10	11.91	2.74	0.72	0.15	3.49	2.98	7.83	1.38	Pull-out
			0	8.61	2.16	0.70	0.12	1.27	1.62	5.53	1.14	Pull-out
		5∅	18	12.71	0.89	0.76	0.01	2.46	0.43	6.33	0.99	Pull-out
			10	13.10	0.33	0.80	0.10	1.45	0.36	5.98	0.38	Pull-out
			0	9.42	0.24	0.82	0.03	1.21	0.28	3.90	0.21	Pull-out
	12	3∅	13	13.29	0.59	1.07	0.19	1.29	0.76	5.09	0.89	Pull-out
			7	10.36	1.23	0.71	0.17	2.60	0.07	6.05	0.63	Pull-out
			0	7.42	0.54	0.31	0.15	1.84	0.06	4.40	0.58	Splitting
		5∅	13	9.52	0.63	1.11	0.12	2.53	0.51	5.31	0.12	Pull-out
			7	9.86	1.09	1.27	0.15	2.01	0.49	5.24	0.70	Pull-out
			0	6.37	0.61	0.11	0.11	2.54	0.43	6.15	0.10	Splitting

slip curves for SCC prism specimens (Fig. 3a, b) and VC prism specimens (Fig. 3c, d), and the column type charts for ultimate bond strengths (Fig. 3e) and bond strengths recorded at a slip of 0.1 mm (Fig. 3f) for prism specimens. In this case the comparison has been made between identical confined and unconfined prism specimens: identical concrete type (SCC or VC), identical test bar diameter (∅10 or ∅12), and identical anchorage length of bar (3∅ or 5∅).

As can be seen in Fig. 3e, the values recorded for the ultimate bond strength (τ_R), were higher for confined specimens up to 55 % (∅12_3∅) for SCC, and up to 59 % (∅12_3∅) for VC. This is probably due to the transverse reinforcement that prevented the propagation of splitting cracks in the plane parallel to the longitudinal axis of the bar, thus increasing the ultimate force at which the bond failure occurs. For the ultimate slip corresponding to the ultimate bond strength (s_u), were recorded very low values for unconfined ∅12 bar diameter specimens due to their splitting failure. For ∅10 bar diameter specimens were recorded higher values for confined specimens up to 142 % (∅10_3∅) in the case of SCC. In the case of VC were recorded higher values for unconfined specimens up to 5 % (∅10_5∅) (Tables 5 and 6). The values obtained for the bond strength recorded at a slip of 0.1 mm ($\tau_{0.1}$), were higher for confined specimens up to 149 % (∅12_3∅) for SCC, and up to 46 % (∅10_3∅) for VC (Fig. 3f). For low level of loads ($\tau_{0.01}$), were recorded higher values for confined specimens up to 96 % (∅12_3∅) for SCC, and up to 160 % (∅10_3∅) for VC (Tables 5 and 6). In Fig. 3b the deviation of the bond stress-slip curve for the specimen SCC ∅12_3∅ _0 % is due to the slower development of the bond strength, as consequence presenting higher slip at ultimate bond strength. Taking into account all these observations and the bond stress-slip curves for SCC prism specimens (Fig. 3a, b), as well as for VC prism specimens (Fig. 3c, d), it can be concluded that the overall bond behavior was better for confined specimens compared to unconfined ones, for all bar diameters (∅10, ∅12) and all anchorage lengths of bar (3∅, 5∅). All the confined specimens had a type of failure by pull-out,

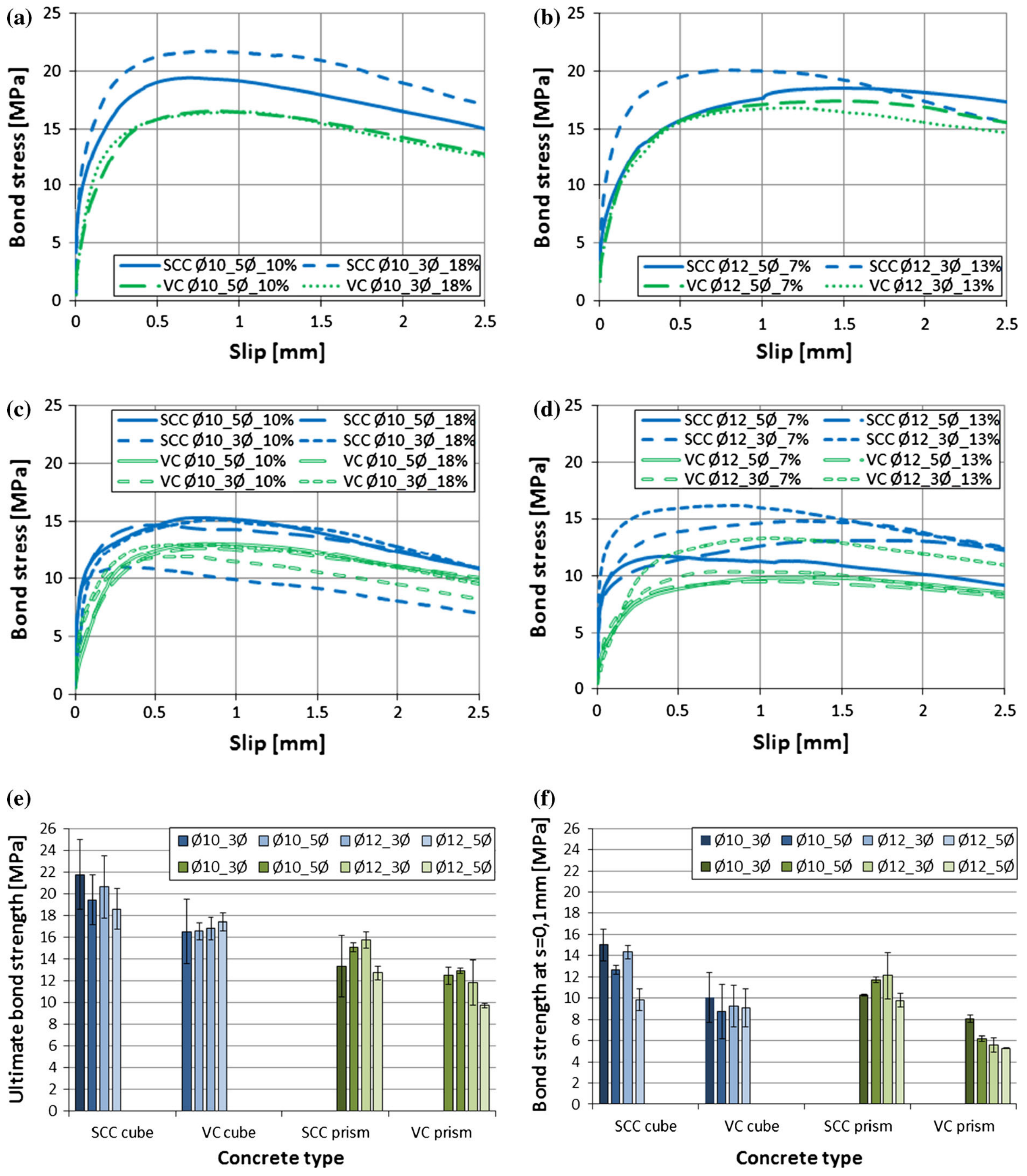

Fig. 2 Influence of the concrete type for confined specimens. **a** Bond stress-slip curves for confined cube specimens with ∅10 test bars, **b** Bond stress-slip curves for confined cube specimens with ∅12 test bars, **c** Bond stress-slip curves for confined prism specimens with ∅10 test bars, **d** Bond stress-slip curves for confined prism specimens with ∅12 test bars, **e** Ultimate bond strengths, **f** Bond strengths at slip $s = 0.1$ mm. Where *∅10_3∅* average value of cube or prism specimens with ∅10 test bar diameter and 3∅ anchorage length of bar, *∅10_5∅* average value of cube or prism specimens with ∅10 test bar diameter and 5∅ anchorage length of bar, *∅12_3∅* average value of cube or prism specimens with ∅12 test bar diameter and 3∅ anchorage length of bar, *∅12_5∅* average value of cube or prism specimens with ∅12 test bar diameter and 5∅ anchorage length of bar

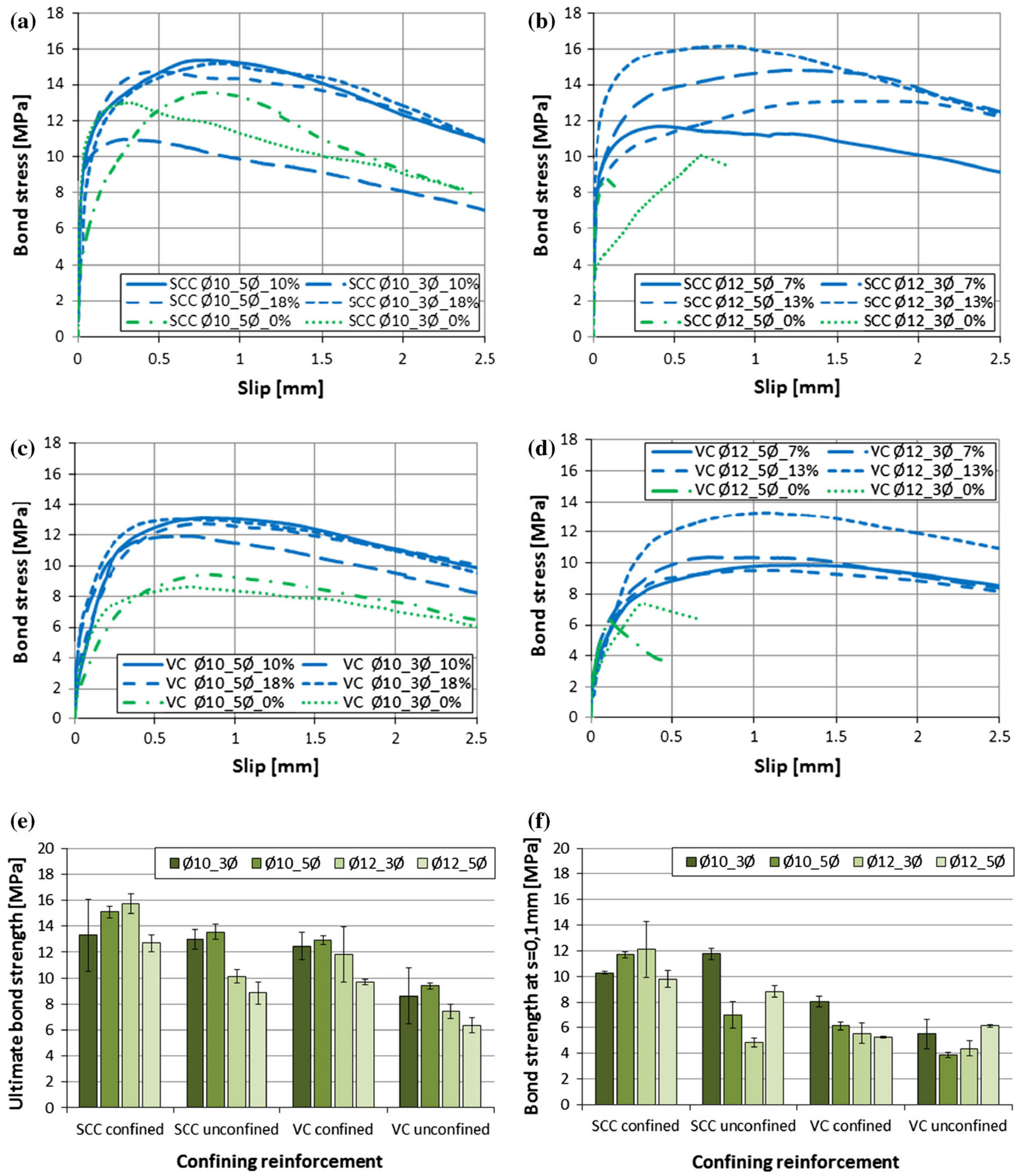

while the unconfined specimens with ∅12 test bar diameter had a splitting failure (Fig. 3b, d); hence the presence of transverse reinforcement changed the type of bond failure from one unfavorable (splitting) to one favorable (pull-out). The bond strength measured when the specimens split is not comparable with the bond at pull-out of bar.

In Fig. 3a–d are presented the bond stress-slip curves for SCC prism specimens and VC prism specimens with different density of transverse

◀**Fig. 3** Influence of the confining reinforcement for prism specimens. **a** Bond stress-slip curves for SCC specimens with ∅10 test bars, **b** Bond stress-slip curves for SCC specimens with ∅12 test bars, **c** Bond stress-slip curves for VC specimens with ∅10 test bars, **d** Bond stress-slip curves for VC specimens with ∅12 test bars, **e** Ultimate bond strengths, **f** Bond strengths at slip s = 0.1 mm. Where *∅10_3∅_10 %* average value of prism specimens with ∅10 test bar diameter, 3∅ anchorage length of bar and 10 % density of transverse reinforcement, *∅10_3∅_18 %* average value of prism specimens with ∅10 test bar diameter, 3∅ anchorage length of bar and 18 % density of transverse reinforcement, *∅10_3∅_0 %* average value of unconfined prism specimens with ∅10 test bar diameter and 3∅ anchorage length of bar, *∅12_5∅_7 %* average value of prism specimens with ∅12 test bar diameter, 5∅ anchorage length of bar and 7 % density of transverse reinforcement, *∅12_5∅_13 %* average value of prism specimens with ∅12 test bar diameter, 5∅ anchorage length of bar and 13 % density of transverse reinforcement, *∅12_5∅_0 %* average value of unconfined prism specimens with ∅12 test bar diameter and 5∅ anchorage length of bar

reinforcement (K_{tr}): 10, 18, 7, and 13 %. In the case of SCC for the ultimate bond strength (τ_R) were recorded higher values up to 35 % (∅10_3∅_18 %) for specimens with $K_{tr} = 18$ %, and up to 7 % (∅12_3∅_13 %) for $K_{tr} = 13$ %. In the case of VC for the ultimate bond strength (τ_R) were recorded higher values up to 9 % (∅10_3∅_18 %) for specimens with $K_{tr} = 18$ %, and up to 28 % (∅12_3∅_13 %) for $K_{tr} = 13$ %. An increase in bond strength with increasing density of transverse reinforcement (K_{tr}) was observed. A sufficient confinement of concrete with reinforcing bars crossing the potential plane of splitting can restrict the propagation and opening of splitting cracks, as well as preventing the bond failure by splitting (Fig. 4).

The different bond behavior that can be observed in Figs. 2, 3 and 5 for similar confined specimens is due to the different density of transverse reinforcement. For example, in the case of specimens with ∅10 test bar diameter (SCC ∅10_3∅) with $K_{tr} = 18$ % and $K_{tr} = 10$ %, the specimens with higher K_{tr} presented higher ultimate bond strength (τ_R). Also, the different bond behavior that can be observed in the case of specimens with ∅12 test bar diameter is due to the same reason, the different density of transverse reinforcement ($K_{tr} = 13$ % and $K_{tr} = 7$ %). The ACI 408R-03 [29] stated that increasing the amount of transverse reinforcement, increases the ultimate bond strength (τ_R), changing the type of bond failure from splitting to pull-out. Meanwhile Orangun et al. [30] argued that an increase of transverse reinforcement over the necessary required to change the type of bond failure, from splitting to pull-out, becomes inefficient because it does not increase the ultimate bond strength. In the pull-out tests carried out for this study it was observed that if the bond failure is caused by splitting, the bond resistance drops rapidly to zero after the occurrence of splitting cracks (Fig. 3b, d). To ensure that the bond failure is caused by pull-out, normally a transverse reinforcement must be provided. The minimum area for this reinforcement is given in Eq. 1.

3.3 Influence of the bar diameter

In order to analyze the influence of test bar diameter, below are presented the bond stress-slip curves for confined prism specimens (Fig. 5a, b), and the column

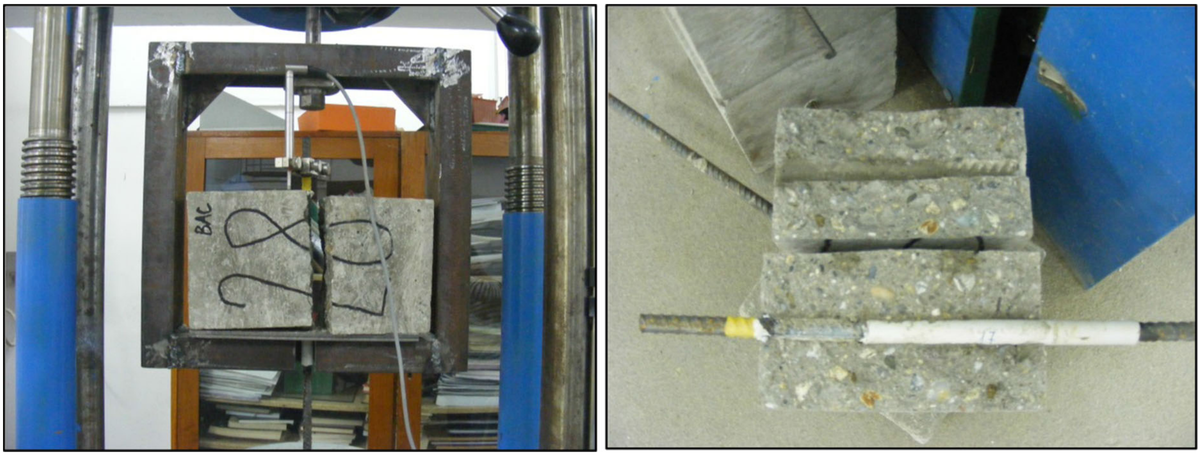

Fig. 4 Pictures from pull-out test, failure of unconfined specimens by splitting

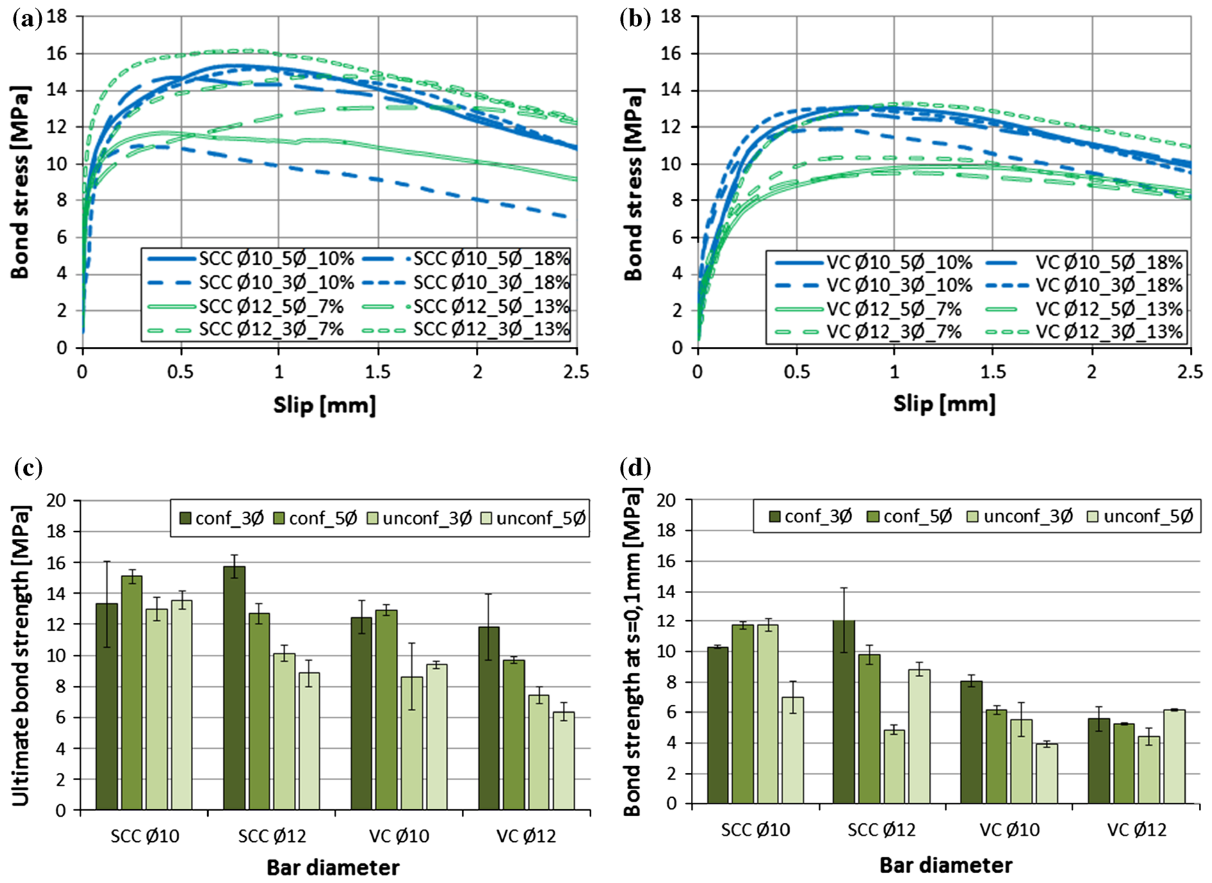

Fig. 5 Influence of the bar diameter for prism specimens. **a** Bond stress-slip curves for SCC specimens, **b** Bond stress-slip curves for VC specimens, **c** Ultimate bond strengths, **d** Bond strengths at slip s = 0.1 mm. Where *conf_3∅* average value of confined prism specimens with 3∅ anchorage length of bar, *conf_5∅* average value of confined prism specimens with 5∅ anchorage length of bar, *unconf_3∅* average value of unconfined prism specimens with 3∅ anchorage length of bar, *unconf_5∅* average value of unconfined prism specimens with 5∅ anchorage length of bar

type charts for ultimate bond strengths (Fig. 5c) and bond strengths recorded at a slip of 0.1 mm (Fig. 5d) for prism specimens. In this case the comparison has been made between identical ∅10 and ∅12 bar diameter confined or unconfined prism specimens: identical concrete type (SCC or VC), and identical anchorage length of bar (3∅ or 5∅).

In the literature [31, 32] is stated that with the increase of bar diameter there is a tendency to diminish the ultimate bond strength. Indeed, the values recorded for the ultimate bond strength (τ_R), were higher for ∅10 bar diameter specimens up to 53 % (unconf_5∅) in the case of SCC, and up to 33 % (conf_5∅) in the case of VC (Fig. 5c). For the ultimate slip corresponding to the ultimate bond strength (s_u), were recorded very low values for unconfined specimens with ∅12 bar diameter due to their splitting failure. For confined specimens were recorded higher values for ∅12 bar diameter up to 37 % (conf_5∅) in the case of SCC, and up to 53 % (conf_5∅) in the case of VC (Tables 5 and 6). The values obtained for the bond strength recorded at a slip of 0.1 mm ($\tau_{0.1}$) and 0.01 ($\tau_{0.01}$), were contradictory for both concrete types, not always ∅10 bar diameter specimens presented higher values (Fig. 5d, Tables 5 and 6). In the previous investigation at Technical University of Cluj-Napoca by Pop et al. [13], the decrease of the normalized ultimate bond strength ($\tau_{R,n}$), when was increased the bar diameter from ∅10 to ∅14, was up to 42 % for SCC, and up to 33 % for VC. For the slip at the maximum bond strength (s_u), the decrease was 49 % for SCC and 52 % for VC, when the bar diameter was increased from ∅10 to ∅14. This tendency to diminish the ultimate bond strength when

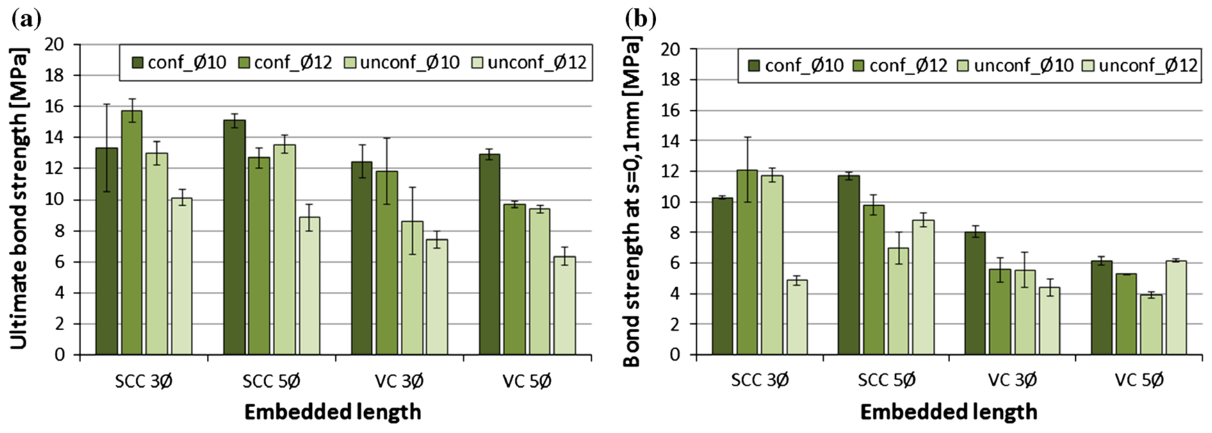

Fig. 6 Influence of the anchorage length of bar for prism specimens, **a** Ultimate bond strengths, **b** Bond strengths at slip s = 0.1 mm. Where *conf_∅10* average value of confined prism specimens with ∅10 test bar diameter, *conf_∅12* average value of confined prism specimens with ∅12 test bar diameter, *unconf_∅10* average value of unconfined prism specimens with ∅10 test bar diameter, *unconf_∅12* average value of unconfined prism specimens with ∅12 test bar diameter

the bar diameter was increased is confirmed by this new experimental study on confined specimens.

3.4 Influence of the anchorage length of bar

In order to analyze the influence of anchorage length of bar, below are presented the column type charts for ultimate bond strengths (Fig. 6a), and bond strengths recorded at a slip of 0.1 mm (Fig. 6b) for prism specimens. In this case the comparison has been made between identical 3∅ and 5∅ anchorage length of bar confined or unconfined prism specimens: identical concrete type (SCC or VC), and identical test bar diameter (∅10 or ∅12).

The values obtained for the ultimate bond strength (τ_R), were higher for 3∅ anchorage length of bar specimens up to 24 % (conf_∅12) in the case of SCC, and up to 22 % (conf_∅12) in the case of VC (Fig. 6a). For the ultimate slip (s_u), were recorded very low values for unconfined specimens due to their splitting failure. For confined specimens with 5∅ anchorage length of bar were recorded higher values up to 29 % (conf_∅12) in the case of SCC, and up to 34 % (conf_∅12) in the case of VC (Tables 5 and 6). The values obtained for the bond strength at a slip of 0.1 mm ($\tau_{0.1}$) and 0.01 ($\tau_{0.01}$), were contradictory for both concrete types, not always the specimens with 3∅ anchorage length of bar presented higher values (Fig. 6b, Tables 5 and 6). In the previous investigation at Technical University of Cluj-Napoca by Pop et al. [13], a decreasing tendency of the ultimate bond strength (τ_R) with increased anchorage length was found. This general tendency was observed in this new experimental study on confined specimens, yet with some exceptions.

3.5 Comparison of test results with other studies and fib Model Code 2010

For short anchorage length ($l_d \leq 5\varnothing$) where a local bond stress-slip relationship is considered, as it was the case in this study, fib MC2010 [19] introduce the following equations for confined concrete for the ultimate bond strength in a pull-out failure (Eq. 3), respectively splitting failure (Eq. 4):

$$\tau_R = 2.5\sqrt{f_{cm}} \tag{3}$$

and

$$\tau_{R,split} = \eta_2 \cdot 6.5 \cdot \left(\frac{f_{cm}}{25}\right)^{0.25} \cdot \left(\frac{25}{\varnothing}\right)^{0.2} \cdot \times \left[\left(\frac{c_{min}}{\varnothing}\right)^{0.33} \cdot \left(\frac{c_{max}}{c_{min}}\right)^{0.1} + k_m K_{tr}\right] \tag{4}$$

where f_{cm} is the mean cylinder concrete compressive strength (N/mm^2), η_2 is the 1.0 for good bond conditions, η_2 is the 0.7 for all other bond conditions, ∅ is the diameter of the anchored bar (mm), c_{min} is the minimum concrete cover (mm), c_{max} is the maximum concrete cover (mm), k_m is the confinement coefficient for transverse reinforcement, K_{tr} is the density of transverse reinforcement.

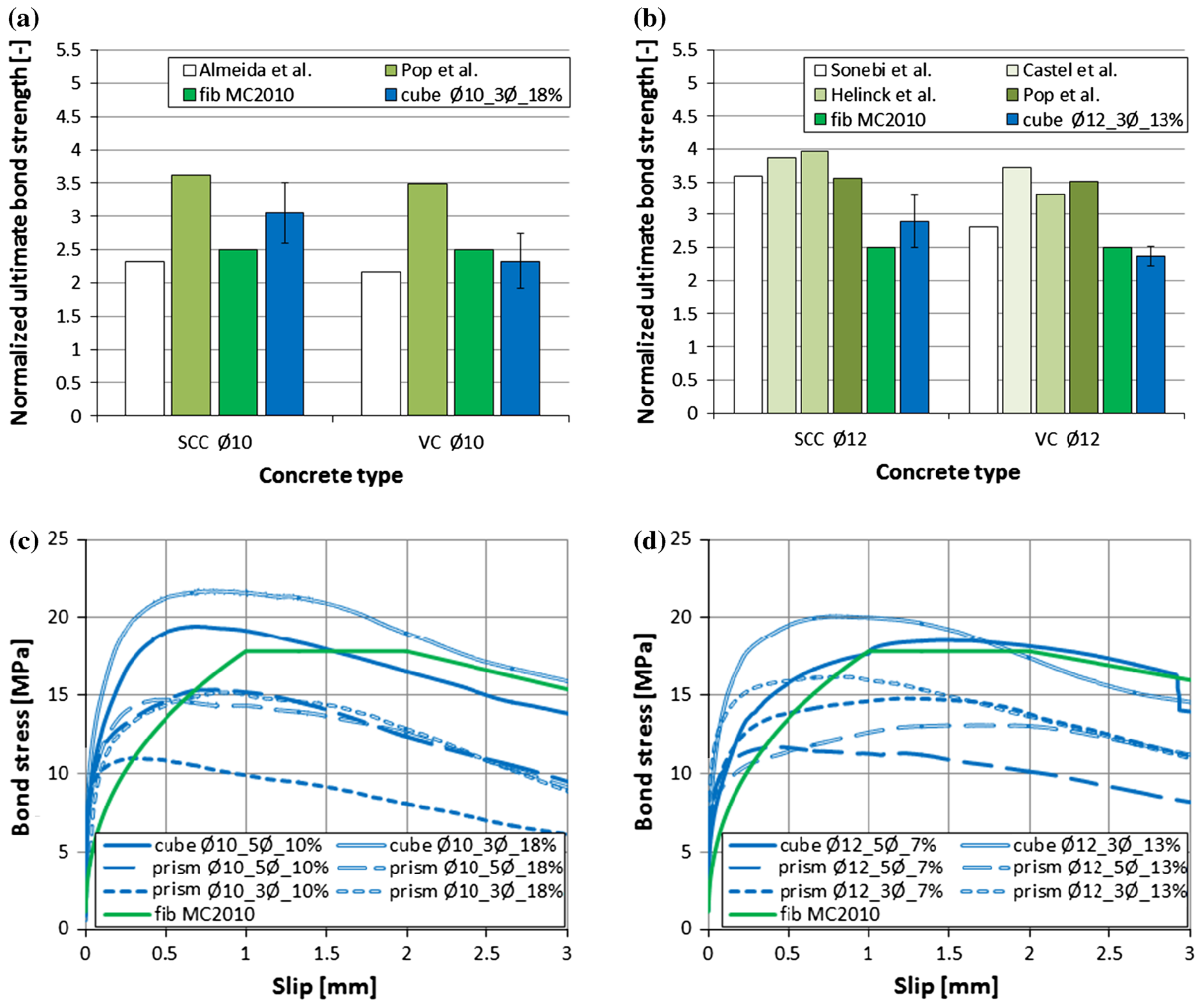

Fig. 7 Comparison of test results with other studies and fib MC2010 [19]. **a** Normalized ultimate bond strengths for specimens with ∅10 test bars, **b** Normalized ultimate bond strengths for specimens with ∅12 test bars, **c** Bond stress-slip curves for SCC specimens with ∅10 test bars, **d** Bond stress-slip curves for SCC specimens with ∅12 test bars

In order to make a comparison with other studies from the literature, the results for the ultimate bond strength (τ_R) are normalized (Eq. 5) to exclude the influence of the tensile strength, which can be correlated to the compressive strength since in normal strength concrete ($f_{ck} \leq 50$ MPa) the tensile strength depend on compressive strength by means of a power-type law (EC2 [33]).

$$\tau_{R,n} = \frac{\tau_R}{\sqrt{f_c}} \tag{5}$$

The test results for cube specimens are compared with other studies from pull-out tests in Fig. 7a for ∅10 test bars and in Fig. 7b for ∅12 test bars. The studies for which were calculated the normalized ultimate bond strength are: Almeida et al. [8]; Pop et al. [13]; Sonebi et al. [5]; Castel et al. [34], and Helinck et al. [32]. Figure 7a, b show once more the better bond strength in the case of SCC. Also, the ultimate bond strengths according to fib MC2010 [19] (Eq. 3) were calculated for the concrete strengths obtained in this study, after which the results were normalized and included in Fig. 7a, b. The values obtained in this study are generally lower than those obtained in other studies, being close to those indicated by fib MC2010 [19] for pull-out failure and good bond conditions.

In Fig. 7c, d are presented the bond stress-slip curves from test results, and the bond stress-slip curve from fib MC2010 [19] calculated for the same

concrete strengths and test bar characteristics that were used in this study. For SCC specimens it can be seen that the bond model for pull-out failure from fib MC2010 [19] underestimate the ultimate bond strength (τ_R) for SCC cube specimens, and overestimate it for SCC prism specimens.

4 Proposed analytical bond model for pull-out failure

A new analytical bond model for pull-out failure was developed based on the test results. We chose to propose new equations and not to develop these equations based on those already existing in the literature because the majority of existing equations are based on splitting failure, whereas in this study the bond failure was by pull-out. The main bond stress-slip curve of the proposed model is similar to fib MC2010 [19] model, but the ultimate bond strength (τ_R) for pull-out failure (Eq. 3) is different. Equation 3 considers only the concrete properties (compressive strength) for the calculation of the ultimate bond strength. The proposed equations from this study consider also the reinforcing bar properties (relative rib area), the anchorage length of bar, the concrete cover, and the confining reinforcement, as these parameters contributed to the ultimate bond strength. The relationship proposed for the ultimate bond strength (τ_R) is based on regression analysis using the experimental data from this study, with the results expressed as Eqs. 6 and 7. In these equations the influence of the following parameters is considered: anchorage length of bar ($\varnothing/l_d$), relative rib area (a_{max}/c), concrete cover ($c_{min}/\varnothing$), density of transverse reinforcement (K_{tr}), and concrete compressive strength (f_{cm}).

For SCC

$$\tau_R = \left(1.03 \cdot \frac{\varnothing}{l_d} + 21 \cdot \frac{a_{max}}{c} + 0.10 \cdot \frac{c_{min}}{\varnothing} + 2.55 \cdot K_{tr} - 1.14\right) \cdot f_{cm}^{0.55} \tag{6}$$

For VC

$$\tau_R = \left(0.17 \cdot \frac{\varnothing}{l_d} + 49.96 \cdot \frac{a_{max}}{c} + 0.09 \cdot \frac{c_{min}}{\varnothing} + 3.67 \cdot K_{tr} - 3.68\right) \cdot f_{cm}^{0.5} \tag{7}$$

where $\varnothing$ is the diameter of the anchored bar (mm), l_d is the anchorage length of bar (mm), a_{max} is the height of transverse ribs (mm), c is the distance between the transverse ribs (mm), c_{min} is the minimum concrete cover (mm), K_{tr} is the density of transverse reinforcement (-), f_{cm} is the mean cylinder concrete compressive strength (N/mm^2).

Equations 6 and 7 are valid for normal strength concrete ($f_{ck} \leq 50$ MPa) with concrete cover $c_{min} \geq 3\varnothing$ and heavy transverse reinforcement ($K_{tr} \geq 0.07$). These equations have been derived from pull-out tests on bars with short anchorage length ($l_d \leq 5\varnothing$) and high relative rib area ($f_R \geq 0.090$).

According to EC2 [33] the concrete tensile strength (f_{ct}) is proportional to the compressive strength (f_{ck}) to the $^2\!/_3$ power for normal strength concrete ($f_{ck} \leq 50$ MPa). fib MC2010 [19] considers the bond strength as proportional to the square root of compressive strength. Taking into account the relationship between tensile and compressive strength we may conclude that bond strength is proportional to the tensile strength to the $^3\!/_4$ power. In the proposed bond model for VC (Eq. 7) it was considered the same influence of the tensile strength on bond strength as in fib MC2010 [19]. An important observation is that the tensile strength of SCC which was 30 % higher than that of VC, despite the fact that SCC and VC had the same compressive strength, increased the bond strength of SCC compared to VC. Because of this fact in the proposed bond model the influence of the concrete tensile strength on bond strength is higher for SCC and is proportional to ($f_{cm}^{0.55}$), as proposed in a recent study by Aslani and Nejadi [35]. The anchorage length of bar (l_d) and the concrete cover (c_{min}) are important parameters that affect the bond strength. Authors like Orangun et al. [30], Chapman and Shah [36], and Harajli [37] consider these parameters in the bond strength equation by the ratio between the bar diameter ($\varnothing$) and the anchorage length (l_d), respectively the ratio between the minimum concrete cover (c_{min}) and the bar diameter ($\varnothing$). In this experimental study the ratio $\varnothing/l_d$ was 0.33 for 3$\varnothing$ anchorage length and 0.20 for 5$\varnothing$ anchorage length, and the ratio $c_{min}/\varnothing$ was 3.00 for prism specimens, and 7.80 and 9.50 for cube specimens. According to ACI 408R-03 [29] the relative rib area of the bar (f_R) is approximately equal to the ratio between the height of transverse ribs (a_{max}) and the distance between the transverse ribs (c). In this

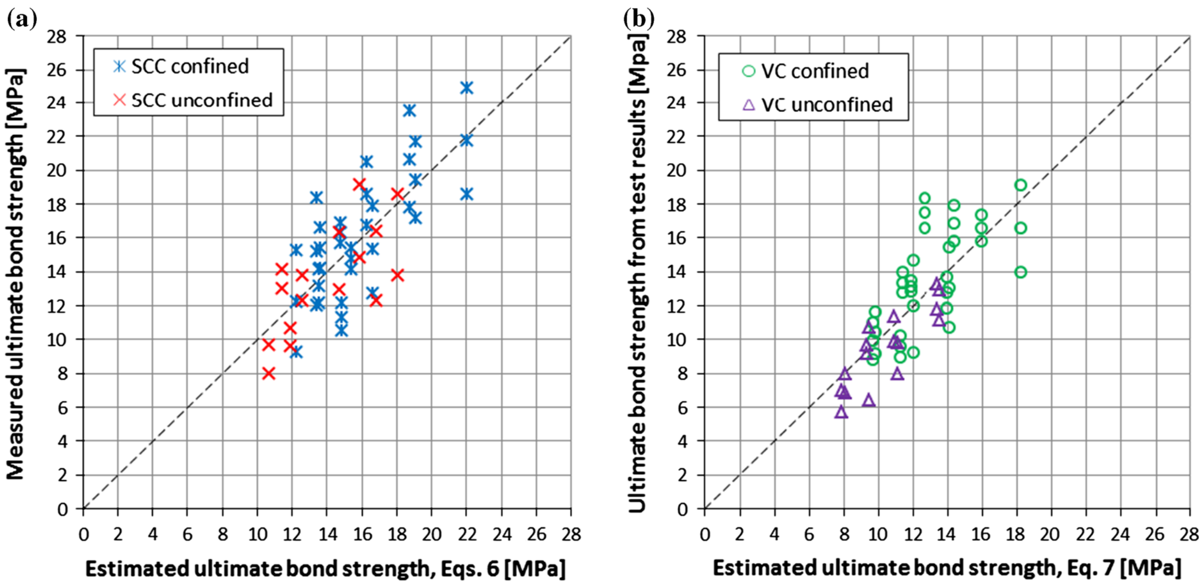

Fig. 8 Comparison between estimated bond strength and measured values. **a** SCC bond strengths estimated by Eq. 6 versus measured values, all results, **b** VC bond strengths estimated by Eq. 7 versus measured values, all results

experimental study the ratio was 0.094 for ∅10 test bars, and 0.090 for ∅12 test bars. The relative rib area was not included as a parameter in the bond model from fib MC2010 [19] (Eq. 4) despite the fact that a recent study by Metelli and Plizzari [17] reported an increase of 40 % in bond strength with an increase in relative rib area from 0.04 to 0.10. In the recent fib Bulletin 72 [38] it is argued that by using specimens with short bond length and high concrete cover, the results obtained by the authors are not considered relevant to splitting failure, as the bond model from fib MC2010 [19] is based on splitting failure. In the proposed bond model from this study which is based on pull-out failure, the relative rib area was included as a parameter. Bond strength equations of laps and anchorages that consider the confinement by transverse reinforcement have been provided by Zuo and Darwin [39], by Canbay and Frosch [40], and by Esfahani and Kianoush [41]. In this experimental study the density of transverse reinforcement (K_{tr}) was 0.10 and 0.18 for ∅10 test bars, respectively 0.07 and 0.13 for ∅12 test bars.

The coefficients in Eqs. 6 and 7 have been calibrated against the experimental data from this study and are empirically derived to fit this data. The experimental data includes test results for similar confined and unconfined specimens as follows: 72 confined specimens and 32 unconfined specimens. The data was also checked for any significant cross-correlations, but none was found. Equations 6 and 7 have been calibrated taking into account the influence of confining reinforcement from the outset. The comparison between estimated and measured bond strengths is shown in Figs. 8a for SCC and in Fig. 8b for VC. Figure 8a, b show that Eqs. 6 and 7 provide a reasonable representation of experimental data from both SCC and VC test results. The equations provide a prediction of bond strength test results with a coefficient of correlation factor (R^2) of 0.58 for SCC, and 0.62 for VC.

The statistical fit of bond strengths estimated by Eqs. 6 and 7 to experimental data for SCC and VC is summarized in Table 7, based on the ratio of bond strength measured in tests to that calculated by Eqs. 6 and 7. The 5 % characteristic value in Table 7 was obtained from a frequency distribution for all measured/estimated bond strength ratios. The validity of Eqs. 6 and 7 was also verified by examining the influence of each parameter on the ratio of measured/estimated bond strength, the graph trend of the data being horizontal.

5 Conclusions

An extensive experimental program on local bond stress-slip relationship of specimens with short anchorage length ($l_d \leq 5∅$) was conducted. A total

Table 7 Summary statistical data for Eqs. 6 and 7

	SCC confined	SCC unconfined	SCC all results	VC confined	VC unconfined	VC all results
Mean	1.02	0.96	1.00	1.04	0.91	1.00
Standard deviation	0.157	0.160	0.158	0.169	0.126	0.167
Coefficient of variation	0.154	0.167	0.158	0.163	0.138	0.167
Minimum	0.71	0.73	0.71	0.75	0.68	0.68
5 % characteristic value	0.76	0.74	0.75	0.76	0.71	0.74
No. of results	36	16	52	36	16	52

of 104 specimens, 40 cubes and 64 prisms, were cast using SCC and VC as follows: series of three identical confined specimens and series of two identical unconfined specimens. The test results were used to analyze the effect of concrete type (SCC and VC), confining reinforcement (confined and unconfined specimens), test bar diameter (∅10 and ∅12), and anchorage length of bar (3∅ and 5∅). From the parametric analysis the following conclusions can be drawn:

- SCC presented better bond behavior than VC in terms of the ultimate slip (up to 25 % lower) and all the bond strengths: ultimate bond strength (up to 32 % higher), bond strength recorded at a slip of 0.1 mm (up to 117 % higher), and bond strength recorded at a slip of 0.01 mm (up to 266 % higher).
- The confining of concrete with transverse reinforcement lead to the increase of all the bond strengths despite of the concrete type, as follows: ultimate bond strength (up to 55 % increase), bond strength recorded at a slip of 0.1 mm (up to 149 % increase), and bond strength recorded at a slip of 0.01 mm (up to 96 % increase).
- A decreasing tendency of the ultimate bond strength with increasing reinforcing bar diameter was observed despite of the concrete type. The specimens with ∅10 test bar presented up to 53 % higher ultimate bond strength than similar specimens with ∅12 test bar.
- In general a decreasing tendency of the ultimate bond strength with increasing anchorage length was found despite of the concrete type, yet with some exceptions. The specimens with 3∅ anchorage length of bar presented up to 24 % higher ultimate bond strength than similar specimens with 5∅ anchorage length of bar.
- The proposed bond model for pull-out failure based on regression analysis of the experimental data from this study considers the favorable effects that the confining reinforcement and relative rib area have on bond strength, making this model more suitable that the existing model from fib MC2010 which considers only the concrete compressive strength.
- The tensile strength of SCC was 30 % higher than that of VC, despite the fact that SCC and VC had the same compressive strength. The higher tensile strength of SCC lead to increased bond strength of SCC compared to VC. Because of this fact in the proposed bond model for SCC the influence of the concrete tensile strength on bond strength is considered higher, and is proportional to ($f_{cm}^{0.55}$).
- The minimum anchorage length for design with SCC can be reduced compared to VC based on the 32 % higher bond strength of SCC obtained in this study. This reduction cannot be linearly related to bond strength due to the nonlinear relationship between the anchorage length and the bond strength.

The proposed bond strength equations (Eqs. 6 and 7) are promising to predict the bond strength between concrete and reinforcement, and can be used to determine the anchorage lengths of reinforcing bars in well-confined concrete where a pull-out bond failure takes place. Nevertheless, further validation and calibration of the model are required using other test results on different concrete grades and reinforcing bars geometries.

Acknowledgments This paper was supported by the project "Improvement of the doctoral studies quality in engineering science for development of the knowledge based society-QDOC" contract no. POSDRU/107/1.5/S/78534, project co-funded by the European Social Fund through the Sectorial

Operational Program Human Resources 2007-2013. The authors gratefully acknowledge professor Carlos A. Gaviria for his feedback in the preparation of this paper.

References

1. Okamura H, Ouchi M (2003) Self-Compacting Concrete. J Adv Concr Technol 1:5–15. doi:10.3151/jact.1.5
2. Mata LA (2005) Implementation of self-consolidating concrete (SCC) for prestressed concrete girders. Department of Civil, Construction, and Environmental Engineering, North Carolina State University, Raleigh, NC, 27695-7908
3. Gibbs JC, Zhu W (1999) Strength of hardened self compacting concrete. In: 1st international RILEM symposium on self-compacting concrete. pp 199–209
4. Sonebi M, Bartos PJM (1999) Hardened SCC and its bond with reinforcements. In: 1st international RILEM symposium on self-compacting concrete. pp 275–289
5. Sonebi M, Bartos P, Zhu W, et al. (2000) Properties of hardened concrete, Task 4, final report. Advance Concrete Masonry Center, University of Paisley
6. Collepardi M, Borsoi A, Collepardi S, Troli S (2005) Strength, shrinkage and creep of SCC and flowing concrete. In: 4th international RILEM symposium on self-compacting concrete. pp 911–919
7. Domone PL (2007) A review of the hardened mechanical properties of self-compacting concrete. Cem Concr Compos 29:1–12. doi:10.1016/j.cemconcomp.2006.07.010
8. de Almeida Filho FM, El Debs MK, El Debs ALHC (2008) Bond-slip behavior of self-compacting concrete and vibrated concrete using pull-out and beam tests. Mater Struct 41:1073–1089. doi:10.1617/s11527-007-9307-0
9. Hossain KMA, Lachemi M (2008) Bond behavior of self-consolidating concrete with mineral and chemical admixtures. J Mater Civ Eng 20:608–616. doi:10.1061/(ASCE)0899-1561(2008)20:9(608)
10. Valcuende M, Parra C (2009) Bond behaviour of reinforcement in self-compacting concretes. Constr Build Mater 23:162–170. doi:10.1016/j.conbuildmat.2008.01.007
11. Desnerck P, De Schutter G, Taerwe L (2010) Bond behaviour of reinforcing bars in self-compacting concrete: experimental determination by using beam tests. Mater Struct 43:53–62. doi:10.1617/s11527-010-9596-6
12. Hassan AAA, Hossain KMA, Lachemi M (2010) Bond strength of deformed bars in large reinforced concrete members cast with industrial self-consolidating concrete mixture. Constr Build Mater 24:520–530. doi:10.1016/j.conbuildmat.2009.10.007
13. Pop I, De Schutter G, Desnerck P, Onet T (2013) Bond between powder type self-compacting concrete and steel reinforcement. Constr Build Mater 41:824–833. doi:10.1016/j.conbuildmat.2012.12.029
14. Schiessl A, Zilch K (2001) The effect of the modified composition of SCC on shear and bond behavior. In: 2nd international RILEM symposium on self-compacting concrete. pp 501–506
15. Almeida Filho FM, De Nardin S, El Debs ALHC (2005) Evaluation of the bond strength of self-compacting concrete in pull-out tests. In: 4th international RILEM symposium on self-compacting concrete. pp 953–958
16. Rehm G (1969) Evaluation criteria for high-bond rebars (in German). Festschrift Rusch
17. Metelli G, Plizzari GA (2014) Influence of the relative rib area on bond behaviour. Mag Concr Res 66:277–294. doi:10.1680/macr.13.00198
18. Darwin D, McCabe SL, Idun EK, Schoenekase SP (1992) Development length criteria: bars not confined by transverse reinforcement. ACI Struct J 89:709–720. doi:10.14359/4158
19. fib (2013) fib Model Code for Concrete Structures 2010. Wiley-VCH Verlag GmbH & Co. KGaA, Weinheim
20. Bažant ZP, Li Z, Thoma M (1995) Identification of stress-slip law for bar or fiber pullout by size effect tests. J Eng Mech 121:620–625. doi:10.1061/(ASCE)0733-9399(1995)121:5(620)
21. Morita S, Fuji S, Kondo G (1994) Experimental study on size effect in concrete structures. In: Mihashi H, Okamura H, Bazant ZP (eds) Size effect in concrete structures: proceedings of the Japan Concrete Institute International Workshop, Sendai, Japan. E & FN Spon, London, pp 27–46
22. CEN (2011) BS EN 197-1:2011. Cement. Composition, specifications and conformity criteria for common cements. British Standards Institution
23. European Project Group (2005) The European guidelines for self-compacting concrete: specification, production and use. EFNARC
24. CEN (2012) BS EN 12390-1. Testing hardened concrete. Shape, dimensions and other requirements for specimens and moulds. British Standards Institution
25. CEN (2010) BS EN ISO 15630-1. Steel for the reinforcement and prestressing of concrete. Test methods. reinforcing bars, wire rod and wire. British Standards Institution
26. Eligehausen R, Popov EP, Bertero VV. (1983) Local bond stress-slip relationships of deformed bars under generalized excitations. University of California, Berkeley, CA, USA, Report No. UCB/EERC-83/23
27. RILEM (1994) AAC 8.1 Pull-out test for reinforcement. RILEM recommendations for the testing and use of construction mater
28. Khayat KH (1998) Use of viscosity-modifying admixture to reduce top-bar effect of anchored bars cast with fluid concrete. ACI Mater J 95:158–167
29. ACI Committee 408 (2003) ACI 408R-03: bond and development of straight reinforcing bars in tension. American Concrete Institute
30. Orangun CO, Jirsa JO, Breen JE (1977) A reevaluation of test data on development length and splices. ACI J Proc 74:114–122. doi:10.14359/10993
31. Pozolo A, Andrawes B (2011) Analytical prediction of transfer length in prestressed self-consolidating concrete girders using pull-out test results. Constr Build Mater 25:1026–1036. doi:10.1016/j.conbuildmat.2010.06.076
32. Helincks P, Boel V, De Corte W et al (2013) Structural behaviour of powder-type self-compacting concrete: bond performance and shear capacity. Eng Struct 48:121–132. doi:10.1016/j.engstruct.2012.08.035
33. EN 1992-1 (2004) Eurocode 2: Design of concrete structures—Part 1-1: General rules and rules for buildings. CEN
34. Castel A, Vidal T, Viriyametanont K, Francois R (2006) Effect of reinforcing bar orientation and location on bond

with self-consolidating concrete. ACI Struct J 103:559–567. doi:10.14359/16432
35. Aslani F, Nejadi S (2012) Bond behavior of reinforcement in conventional and self-compacting concrete. Adv Struct Eng 15:2033–2052. doi:10.1260/1369-4332.15.12.2033
36. Chapman RA, Shah SP (1987) Early-age bond strength in reinforced concrete. ACI Mater J 84:501–510
37. Harajli MH (1994) Development/splice strength of reinforcing bars embedded in plain and fiber reinforced concrete. ACI Mater J 91:511–520
38. Cairns J (2014) Bond and anchorage of embedded reinforcement: Background to the fib Model Code for Concrete Structures 2010: Technical report. International Federation for Structural Concrete (fib)
39. Zuo J, Darwin D (2000) Splice strength of conventional and high relative rib area bars in normal and high-strength concrete. ACI Struct J 97:630–641
40. Canbay E, Frosch RJ (2005) Bond strength of lap-spliced bars. ACI Struct J 102:605–614. doi:10.14359/14565
41. Esfahani MR, Kianoush MR (2005) Development/splice length of reinforcing bars. ACI Struct J 102:22–30. doi:10.14359/13527
